# Excess Rab4 rescues synaptic and behavioral dysfunction caused by defective HTT-Rab4 axonal transport in Huntington’s disease

**DOI:** 10.1186/s40478-020-00964-z

**Published:** 2020-07-01

**Authors:** Joseph A. White, Thomas J. Krzystek, Hayley Hoffmar-Glennon, Claire Thant, Katherine Zimmerman, Gary Iacobucci, Julia Vail, Layne Thurston, Saad Rahman, Shermali Gunawardena

**Affiliations:** 1 Department of Biological Sciences, The State University of New York at Buffalo, New York, 14260 USA; 2 Department of Biological Engineering, Cornell University, Ithaca, NY USA

**Keywords:** Huntingtin, Rab4, Axonal transport, Synaptic defects, Behavioral deficits, In vivo imaging, *Drosophila*, iPSCs, Huntington’s disease

## Abstract

Huntington’s disease (HD) is characterized by protein inclusions and loss of striatal neurons which result from expanded CAG repeats in the poly-glutamine (polyQ) region of the huntingtin (*HTT)* gene. Both polyQ expansion and loss of HTT have been shown to cause axonal transport defects. While studies show that HTT is important for vesicular transport within axons, the cargo that HTT transports to/from synapses remain elusive. Here, we show that HTT is present with a class of Rab4-containing vesicles within axons in vivo. Reduction of HTT perturbs the bi-directional motility of Rab4, causing axonal and synaptic accumulations. In-vivo dual-color imaging reveal that HTT and Rab4 move together on a unique putative vesicle that may also contain synaptotagmin, synaptobrevin, and Rab11. The moving HTT-Rab4 vesicle uses kinesin-1 and dynein motors for its bi-directional movement within axons, as well as the accessory protein HIP1 (HTT-interacting protein 1). Pathogenic HTT disrupts the motility of HTT-Rab4 and results in larval locomotion defects, aberrant synaptic morphology, and decreased lifespan, which are rescued by excess Rab4. Consistent with these observations, Rab4 motility is perturbed in iNeurons derived from human Huntington’s Disease (HD) patients, likely due to disrupted associations between the polyQ-HTT-Rab4 vesicle complex, accessory proteins, and molecular motors. Together, our observations suggest the existence of a putative moving HTT-Rab4 vesicle, and that the axonal motility of this vesicle is disrupted in HD causing synaptic and behavioral dysfunction. These data highlight Rab4 as a potential novel therapeutic target that could be explored for early intervention prior to neuronal loss and behavioral defects observed in HD.

## Introduction

Huntington’s disease (HD) is a deadly, dominantly-inherited neurodegenerative disorder that is caused by expansion of CAG repeats in the poly-glutamine (polyQ) tract (> 35 repeats) in the huntingtin (*HTT*) gene. Disease progression is characterized by cytoplasmic and nuclear inclusions together with significant loss of striatal neurons resulting in dramatic changes to behavior, including uncontrollable movements (chorea) and cognitive decline [54]. The earliest known dysfunction seen in HD involves changes to synaptic proteins and deficits in synaptic plasticity [52]. These defects could arise due to disruption of long-distance transport within axons. Indeed, axonal transport defects have been observed in HD prior to behavioral defects and neuronal cell death in flies [24], mice [71], and humans [64], indicating that perturbations in trafficking can occur early in the development of HD [24, 27]. Further, HTT moves bi-directionally within axons [7] and reduction of *Drosophila* HTT (htt) causes axonal accumulations [24, 36, 75, 76], similar to what has been observed with loss of motor protein function [23]. Loss of HTT in mammalian neurons also disrupts the transport of brain-derived neurotrophic factor (BDNF), which was partially rescued by the expression of htt, indicating a conserved role for HTT during axonal transport. We previously identified functional interactions between HTT and molecular motors kinesin-1 and dynein [78]. Biochemical analysis also showed associations between HTT and motors. In mammals, HTT associates with dynein intermediate chain [12] and dynactin [40], and with kinesin light chain (KLC) [50] and p150_glued_ (a subunit of dynactin) [16] via interactions with huntingtin-associated protein 1 (HAP1). However, despite growing evidence of a role for HTT in axonal transport, the specific vesicle complexes that HTT is present on, and the cargoes that HTT-containing vesicles carry during long distance axonal motility in vivo remain elusive.

Recent studies suggest that HTT likely functions in conjunction with particular Rab-GTPases during trafficking. HTT immunoprecipitated with Rab11 and influences Rab11 activation [42], while reduction of htt disrupts the axonal transport of Rab11 in vivo [57]. Rab11-dependent vesicle recycling was also perturbed in HD-patient fibroblasts [43]. Intriguingly, expression of Rab11 ameliorated synaptic and behavioral dysfunction seen in a *Drosophila* HD model [60] and rescued neurodegeneration in HD mice [69]. Further, we previously showed that reduction of htt disrupts the bi-directional axonal motility of Rab3 (synaptic vesicles), Rab19 (recycling), the retrograde motility of Rab7 (late endosome/lysosome), and the anterograde motility of Rab2 (ER-Golgi) from 17 neuronal Rabs examined [77]. HTT also co-migrates with all of these Rabs within larval axons [77]. While this work postulates that HTT differentially influences the axonal motility of specific Rab-GTPases in vivo, the role of particular HTT-Rab containing cargo complexes at synapses is unknown.

In this study, we focused on isolating a putative moving HTT-Rab4 vesicle complex. Using a combination of in vitro and in vivo analysis, and a variety of model systems including *Drosophila,* mice, and iNeurons derived from induced pluripotent stem cells (iPSCs) obtained from HD patients, we identified the role of HTT in the axonal motility of Rab4-containing vesicles. In vivo imaging and biochemical evidence indicate the existence of a moving HTT-Rab4 vesicle complex containing the endosomal transport regulatory protein, HIP1 (huntingtin-interacting peotein1), but not HAP1 (huntingtin associated protein 1). Pathogenic polyQ-HTT disrupted the motility of these HTT-Rab4 vesicles in HD iNeurons and in larval axons expressing pathogenic polyQ-HTT, resulting in larval locomotion defects, aberrant synaptic morphology and decreased lifespan of adult flies. Intriguingly, over-expression of Rab4 ameliorated synaptic morphology and behavioral phenotypes mediated by pathogenic polyQ-HTT and led to increased lifespan of adult flies expressing polyQ-HTT. Together our observations implicate dysfunction in the axonal transport of Rab4 as an early event in the progression of HD, causing synaptic and behavioral defects.

## Materials & methods

### *Drosophila* genetics

UAS-Rab4-mRFP or UAS-HTT15Q-mRFP males were crossed with Appl-GAL4 or Appl-GAL4; T(2,3), CyO, TM6B, Tb^1^ / Pin^88k^ virgin females. The chromosome carrying T(2:3),CyO,TM6B,Tb is referred to as B3 and carries the dominant markers, Hu, Tb and CyO. The larval Tb (tubby) marker is used to select larvae of interest. Males that were Appl-GAL4/Y;UAS-Rab4-mRFP/B3, Appl-GAL4/Y;UAS-YFP-Rab4/B3, Appl-GAL4/Y;UAS-HTT15Q-mRFP/B3 were crossed with either *Drosophila* htt-RNAi [2], Df(98E2);CG9990 [81], Rab4^MI10530^ (BDSC), klc^8ex94^/TM6B (Goldstein), robl^k^/B3 (Goldstein), UAS-HTTex1-25Q-eGFP (Perrimon), UAS-APP-YFP [34], UAS-Syt-GFP, UAS-nSyb-GFP/TM3, UAS-Rab11-GFP, UAS-YFP-Rab3, klp64D^k1^/TM3, unc-104^d11204^/CyO, milt^[k04704]^/CyO, hip1^[MI05905]^, nmo^[P1]^/TM6B, nuf^[MB09772]^, or rip11^[KG02485]^/FM7c (BDSC), UAS-rip11-GFP.CT (Ready, BDSC), UAS-HTT16Q (Vitruvean, BDSC) UAS-HTT128Q (Vitruvean, BDSC), UAS-HTTex1-72Q-eGFP (Perrimon), UAS-HTTex1-103Q-eGFP (Perrimon) or UAS-HTT138Q-mRFP (Littleton) virgins. In all cases, non-tubby female 3rd instar larvae were selected. Sibling tubby larvae were evaluated as controls. Reciprocal crossings were also done to confirm observations. A comprehensive list of *Drosophila* strains used in this study can be found in Table S1.

### In-vivo analysis of vesicle motility within whole mount larval axons

Larvae were dissected and immediately imaged under physiological conditions as previously detailed in White et al. [4, 77]. Non-tubby, female larvae were dissected and imaged under physiological conditions in dissection buffer. Motility was visualized in the red or green, channels using a Nikon TE-2000E inverted fluorescence microscope with a Cool Snap HQ cooled CCD camera (Photometrics, Tucson, AZ, USA), and a ProScan II high speed shutter (100 mm/s) (Nikon, Melville, NY, USA). From each larva, four sets of movies at an imaging window frame size of 90 μm at 150 frames were taken from the mid-region of the larva at an exposure of 500 ms using the Metamorph imaging system (Molecular Devices, Sunnyvale, CA, USA). From a total of 10 larvae a set of 40 movies were imaged for each genotype at a spatial resolution of 0.126 μm/pixel. Movies were analyzed using a MATLAB-based particle tracker program as previously detailed [25, 59, 77]. Briefly, a standard data set consisted of four movies, each lasting 75 s span a total time of 6 min recorded for 10 individual animals. A band (5 pixels in thickness) flanking the axon is extracted from each frame. Bands from all frames are pasted top-to-bottom to form a kymograph computationally. Computationally recovered vesicle trajectories were color-coded and overlaid on the kymograph; colors were selected randomly to differentiate crossing trajectories. Truncated vesicle trajectories are excluded for each movie. Full trajectories are analyzed as detailed in Reis et al. 2012 [59] and Gunawardena et al. 2013 [25]. Vesicle trajectories were analyzed to obtain the overall distribution of cargo populations and individual vesicle movement behaviors (velocities, pause frequencies/durations, run lengths) as previously done [25, 59, 77]. As detailed in Reis et al. 2012 [59] and Gunawardena et al. 2013 [25], the anterograde (retrograde) duration-weighted segmental velocity of a cargo within its track is defined as the sum of its anterograde (retrograde) segmental velocities weighted by their durations and divided by the sum of the durations. Pause frequency details the total number of times a cargo pauses divided by its total time in movement. Pause duration evaluates the total time a cargo pauses within its directional track. Run length is a description of the total distance a cargo moves in a particular direction before pausing or changing direction.

### Simultaneous dual-color in vivo imaging and co-migration analysis in whole mount larval axons

Simultaneous dual-view imaging was done as detailed previously in Banerjee et al. 2020 [4]. Briefly, we used a NikonTE-2000E inverted fluorescence microscope with a beam splitter containing narrow single-band GFP/DsRED filters, a Cool Snap HQ cooled CCD camera, and a ProScan II high speed shutter (100 mm/s) for simultaneous imaging. Movies were taken in Dual-View mode using the split view software in Metamorph at 150 frames from the mid-region of the larvae at an exposure of 500 ms to simultaneously image RFP- and GFP- tagged vesicles. We routinely checked for bleed through by imaging each fluorophore with the individual filters, As seen in Fig. 2 not all fluorophores overlap. In some movies none of the red or green tracks overlap (Rab4-mRFP and YFP-Rab3, Rab4-mRFP and APP-YFP) indicating the specificity of our system. The Cool Snap HQ camera Dual-View mode was aligned using Metamorph software (Split-View settings) before each imaging session. Movies were split by wavelength and each kymograph for each split movie was created, merged and analyzed for co-migration. Trajectories of vesicles with co-localized tracks were identified from kymographs using Metamorph software. For each fluorescence channel, a kymograph was generated using Metamorph as previously done [25, 59, 77]. Briefly, after selecting the first channel, all frames within the time-lapse image sequence of this channel were added together to produce a summed image. To identify vesicles with co-localized signals from both channels, the kymographs were colored in red and green, respectively, and combined into a single RGB kymograph. Note that to differentiate meaningful co-localization we evaluated the co-localization of the entire trajectory of a moving particle during the entire time frame of the movie. Therefore, only particles containing the same trajectory in both red and green would show co-localization in yellow when merged and spurious co-localization observed in a one-time frame would be avoided. The total number of co-localized full trajectories for 10 kymographs across five larvae were counted for each genotype. Pearson’s correlation coefficients, percentage of co-localization between red and green trajectories, and two-color 2D intensity histograms were obtained using Coloc2 and EZ colocalization in ImageJ. Briefly, kymographs from *Drosophila* larval segmental nerves expressing two fluorophores were separated into red and green channels using Metamorph and analyzed using Coloc2 and EZ colocalization in ImageJ.

### Immunohistochemistry in *Drosophila* larval axons and NMJs

Third instar *Drosophila* larvae were dissected and fixed in 4% paraformaldehyde. KLC (Goldstein, 1:100), DIC (Abcam 1:50), or DCSP-3 (DSHB, 1:50) antibodies were used in conjunction with secondary antibodies anti-mouse or anti-rabbit AlexaFlour®488, AlexFlour®568, or Alexaflour®647 (ThermoFisher, 1:100). Images of fixed larval segmental nerves were taken at 60X or 100x using FITC, TxRed, and Cy5 filters which were merged into a single RGB image to analyze co-localization noted as yellow or white puncta. Axonal blockages were quantified as previously done in Gunawardena et al. [24]. For each genotype, a minimum of 5 confocal optical images across 6 larvae were imaged. Sub-pixel imaging refers to the subpixel detection accuracy of fluorescent puncta, which was previously confirmed by directly comparing Gaussian fitting of conventional microscopy data analyzed to super resolution imaging in [73]. Szpankowski et al., 2012 [73] showed that the same subpixel localization method implemented predicted coordinates of detected puncta accurately compared with structured illumination-based OMX (Applied Precision Instruments) super resolution analysis of the same data. For NMJ analysis, HRP-FITC or HRP-TxRED (Jackson ImmunoResearch Labs) was used (1:50). Non-tubby, female larvae were dissected, fixed, and stained with HRP. Quantification of NMJ morphology and Rab4 accumulations were performed as in Kang et al. [34]. We examined type-1 synaptic boutons between muscles 6/7 at larval abdominal segments A4-A5 of third instar larval. Images of NMJs were collected using a Nikon Eclipse TE 2000 U microscope at 60X (Nikon, Melville, NY, USA). For each genotype, a minimum of 4 optical images across 8 larvae were imaged. Bouton number (#), bouton area (μm^2^), and total NMJ length (μm) were measured using NIH ImageJ software. Comprehensive list of antibodies used in this study can be found in Table S1.

### Quantification of *Drosophila* larval motility and adult lifespan

Larval velocity and contractions were performed by visualizing third instar larval crawling patterns on 1% agarose gel, dyed blue for added contrast, that was embedded with a 0.25 cm × 0.25 cm grid. Once placed at the center of the agarose gel, a 2-min interval recording began. Each larva was recorded during three independent trials, controlling for temperature (~ 25 °C) and humidity (~ 60%). Twenty larvae were tested per genotype. Quantifications of the number of contractions and the larval velocity were measured using NIH ImageJ software. A total of 1.5 min of the 2-min recording was utilized for quantification purposes allotting the initial 15 s for larva self-adjustment after being placed in the center of the agarose gel. Lifespan analysis was performed by placing three vials of 20 female adult flies for each genotype at 25 °C, 60% humidity. Flies were counted every day for ~ 70 days to tally the number of survivors.

### WT and HD iPSC cultures and neuronal differentiation

The following cell lines were obtained from the NIGMS Human Genetic Cell Repository at the Coriell Institute for Medical Research: iPCS from WT (GM23279-polyQ = 25, 26y, female) and HD (GM23225-polyQ = 72, 20y, female) patients were purchased from the Coriell Cell Repository (Camden, NJ) (Table S1). iPSCs from WT (ND38555-polyQ = 17, 48y, female) and HD (ND42222-polyQ = 109, 9y, female) patients were purchased from the NINDS Repository (Table S1). iPSCs were grown and expanded on corning matrigel (Fisher) using E8 iPSC media (Invitrogen). Pluripotency was analyzed using an antibody against OCT-3/4 (Santa Cruz, 1:200) and Hoechst was used as a nuclear staining as detailed below. After 4 passages iPSCs were differentiated into neuronal precursors (NPCs) using PSC neural induction media (Invitrogen) and published protocols (publication #MAN0008031). NPCs were identified using an antibody against Nestin (Santa Cruz, 1:200) and then differentiated into mature iNeurons using neurobasal media supplemented with 1X B27 and 2 mM glutamine (Invitrogen, ThermoFisher). Differentiated neurons, identified using antibodies against MAP2 (BD Biosciences, 1:200), βIII-tubulin (Biolegend, 1:200) and Tyrosine Hydroxylase (EMD Millipore, 1:200) exhibited an extensive neurite network after 21 days at which time they were used for electrophysiology experiments, biochemical experiments, immunofluorescence, and transfections.

### Electrophysiology in iNeurons

Whole-cell patch clamp was used to record from cells with neuronal morphology. Borosilicate glass pipettes (World Precision Instruments, Inc., USA, 4–9 MΩ) were filled with (in mM): 135 k-gluconate, 7.5 KCl, 10 phosphocreatine, 10 HEPES, 2 MgATP, 0.3 Na2GTP, pH 7.3 with KOH and adjusted to 290 mOsmol using sucrose. During recording, cells were bathed in (in mM): 140 NaCl, 4 KCl, 2 CaCl2, 2 MgCl2, 10 HEPES, 10 D-glucose, 10 sucrose, pH 7.4 with NaOH. To record voltage-gated Na+/K+ channel activity, a series of depolarizing potentials from -80 mV to 80 mV were applied in 20 mV intervals for 500 msec. Series resistance, whole-cell capacitance, and pipette capacitance were compensated to minimize transient capacitive current artifacts during recording. To inhibit currents, either 100 μM tetrodotoxin (TTX) or 50 mM tetraethylammonium chloride (TEA) was added to the bath via pipette. Currents were low-pass filtered at 2 kHz (Axopatch 200B, 4-pole Bessel) and sampled at 5 kHz (Digidata 1440A). To record action potentials, cells were current-clamped to maintain a holding membrane potential of -65 mV. Cells requiring more than 100pA current injection to maintain -65 mV were discarded. A series of stepwise pulses of current were injected from 0 to + 140 pA in 10pA intervals. Membrane potentials were low-pass filtered at 10 kHz (MultiClamp 700B Commander) and sampled at 20 kHz (Digidata 1440A). All data was recorded in Clampex 10.5 and analyzed in Clampfit 10.2 (Molecular Devices). Voltage-gated Na + channel and voltage-gated K+ channel currents were quantified by peak and steady-state current values, respectively.

### In vitro analysis of Rab4 motility within WT and HD iNeurons

21-day-old differentiated WT and HD iNeurons were transfected with a mammalian expression vector expressing mCherry-Rab4a-7 (Addgene#55125, Table S1) using Lipofectamine 3000 (Fisher). Two to four days post-transfection, transfected neurons expressing mCherry-Rab4 were imaged at 100x and vesicle motility was imaged, analyzed and quantified as described for in vivo analysis of vesicle motility in whole mount larval axons.

### Immunohistochemistry: human iPSCs/NPCs/iNeurons

WT and HD iPSC/NPC colonies or 21-day old differentiated neurons were washed in PBS pH 7.2 and fixed in 4% paraformaldehyde. Blocking solution (1x PBST with 5% BSA) was added to cells for 60 min prior to antibody incubation. Cells were incubated in primary antibodies, OCT-4 (Santa Cruz 1:200), Nestin (Santa Cruz 1:200), MAP2 (BD Biosciences 1:200) βIII-tubulin (Biolegend 1:200), Tyrosine Hydroxylase (EMD Millipore 1:200), Rab4 (Abcam 1:200), HIP1 (Novus Biological, 1:200), DIC (Abcam, 1:200), or PolyQ (EMD Millipore, 1:200) for 16 h at 4 °C and appropriate secondary antibodies (AlexaFluor® 488 or 568, ThermoFisher) for 1 h at 25 °C. DAPI was used to stain nuclei. Cells were then imaged at 20x-40x (for iPSC and NPC) or 60x-100x (iNeurons). iNeurons were imaged on glass slide bottom dishes (In Vitro Scientific China, D29–14-1-N). As above, fixed images were taken at 100x using DAPI, FITC, TxRed, and Cy5 filters which were merged into a single RGB image to analyze co-localization noted as yellow or white puncta. Comprehensive list of reagents and antibodies used in this study can be found in Table S1.

### Preparation of protein extracts from mouse brains and human iNeurons

Mouse brains (gift from K. Medler, C57BL/6) were dissected and halved. Brains were stored on dry ice and used immediately or stored at -80C for future use. Mouse brains were homogenized in homogenization buffer (10 mM HEPES, pH 7.4, 100 mM K acetate, 150 mM sucrose, 5 mM EGTA, 3 mM Mg acetate, 1 mM DTT) containing a cocktail of protease inhibitors (Roche) and 5 mM EDTA. Mouse brain extracts were then centrifuged at 1000 g for 15 min at 4 °C. The supernatant (PNS) was then used for sucrose gradient fractionation analysis as detailed below or for western blotting. WT and HD iNeurons were manually removed from 12-well plates using ice-cold homogenization buffer (as above) and blended for 30 s on ice using a motorized pestle. Neuronal extracts were then centrifuged at 1000 g for 15 min and the supernatant was analyzed using western blot analysis.

### Sucrose gradient fractionations

PNS samples from mouse brain or human iNeuron extracts were further fractionated into soluble, heavy membrane (P1), and vesicle fractions (VF) by sucrose gradient ultra-centrifugations as previously done [3, 15] using lysis buffer (4 mM HEPES, 320 mM sucrose pH 7.4) containing a phosphatase and protease inhibitor cocktail (Pierce). Briefly, 300ul of PNS was combined with 300ul 62% sucrose and layered onto a sucrose gradient (35, and 8% sucrose) and centrifuged at ~ 100,000 g for 90 mins. The vesicle fraction (VF, 35/8 layer), the soluble fraction and the heavy membrane fractions were removed and used in western blot analysis. 100ul of homogenization buffer was used to dissolve the heavy membrane pellet (P1).

### Co-immunoprecipitation analysis

Isolation of Rab4 vesicles was performed as described [3, 15]. 1000μg of total protein from the mouse or human iNeuron vesicle fraction were incubated with a rabbit monoclonal antibody to Rab4 (Abcam, 1:100) for 1 h at 25 °C. Protein homogenates were then incubated with magnetic beads (Pierce Protein A/G Magnetic Beads) with rotation for 90 min and eluted with Pierce elution buffer (pH 2.8). Eluents were then analyzed by western blot analysis as detailed below. Alternatively, Co-IP was performed on protein homogenates using the Pierce™ Co-Immunoprecipitation Kit. Isolation of Rab4 vesicles using magnetic beads occurred the absence of detergents to preserve vesicular membranes**.** Eluents were then separated by SDS-PAGE and analyzed via western blot.

### SDS-PAGE and Western blot analysis

Mouse or human iNeuron fractions in NuPage LDS sample buffer with 4 mM β-mercaptoethanol were run on 4–12% Bis-Tris gels (Invitrogen) and transferred to nitrocellulose membranes. Blots were blocked using TBST with 5% BSA for 60 mins at 25 °C and incubated with primary antibodies (SYT1 (Phosphosolutions 1:1000), Rab4 (Abcam 1:1000), KIF5C (Goldstein 1:500), DIC (Abcam 1:1000), Actin (ThermoFisher 1:1000), Tubulin (Abcam 1:2000), HTT rabbit polyclonal (Abcam 1:1000), HTT mouse monoclonal (EMD Millipore 1:1000), Hip1 (Novus Biological 1:1000), KDEL (Abcam 1:1000), Golgi (Millipore Sigma 1:1000), Cytochrome C (Santa Cruz 1:1000), HAP1 (Santa Cruz 1:1000), Rab11 (Abcam 1:1000), Rab5 (Abcam, 1:1000), or Syntaxin17 (Juhasz, 1:500) for 16 h at 4 °C. Blots were then incubated with anti-mouse or anti-rabbit secondary HRP-conjugated antibodies (ThermoFisher 1:1000) and imaged using a BioRad Chemi-doc system with Pierce ECL or diluted Femto substrate (1:5 in TBS). Images from 3 to 5 blots were quantified using ImageLab. Comprehensive list of reagents and antibodies used in this study can be found in Table S1.

### Statistical analysis

The statistical analysis used for each experiment is indicated in each figure legend. First power and sample size (n) calculations were performed on minitab18 for each experimental paradigm: comparing 2 means from 2 samples, with two-tailed equality to identify the sample size that corresponds to a power of 0.9 with α = 0.01. For each experiment, a stringent significance threshold of *p* < 0.01 (99% confidence) was used as detailed in White et al. [77]. Using Minitab18 calculation of sample size for a power of 0.9 and α = 0.01, the n-value for each experiment was determined based on the control data (average and standard error) in each case. For western blot quantifications, α = 0.05. The n-value refers to the number of larvae, the number of flies, the number of mouse brain lysate, or the number of induced human neurons. Individual data points for each quantification was averaged for each n and then compared.

To select the appropriate statistical test, data distributions for each transport dynamic analyzed were first checked for normality using the nortest package of R: the Lilliefors test and Anderson–Darling test as previously detailed [25, 59, 77]. Statistical significance of normal distributions was calculated by a two-sample two-tailed Student’s t-test and/or ANOVA while the non-normal segmental velocity distributions were compared using the non-parametric Wilcoxon–Mann–Whitney rank sum test in Excel and Minitab18. The global velocity averages across each larva were found to be normal distributions, as well as the global pause frequencies, pause durations and run lengths across each larva. For motility dynamic quantifications, duration-weighted segmental velocities each larva (total of 4 movies, > 500 particles) were pooled, then the average global dur-weighted segmental velocities from each larva were averaged (total 4 movies, > 500 particles) before statistical analysis as detailed in [25, 59]. Therefore, statistical analysis was performed on global velocities from each larva rather than individual particle velocities (*n* = 10 larvae, 4 movies per larvae). Statistical significance was determined using the two-sample two-tailed Student’s t-test.

For statistical analysis on motility dynamics from iNeurons, analysis was performed on individual particle velocities (*n* = 7, > 250 particles), therefore, the non-parametric Wilcoxon–Mann–Whitney rank sum test was utilized due to non-normality in the data. For immunofluorescence analysis, statistical analysis was performed in Excel and Minitab18 using the two-sample two-tailed Student’s *t*-test/ANOVA. Differences were considered significant at a significance level of *p* < 0.01, which means a 99% statistically significant correlation. Based on the power analysis, quantifications were performed across 5, 8, or 10 larvae. For western blots, quantification analysis was performed using Image Lab software. Data obtained from Image Lab was analyzed in Excel and Minitab18 using two-tailed Student’s *t*-test/ANOVA across three independent experiments (*n* = 3). Differences for western blots were considered significant at a significance level of *p* < 0.05. Overlaid dot plots were constructed for all figures using OriginLab / OriginPro.

## Results

### Reduction of HTT disrupts the axonal motility of Rab4 causing axonal and synaptic Rab4 accumulations

**Fig. 1:**
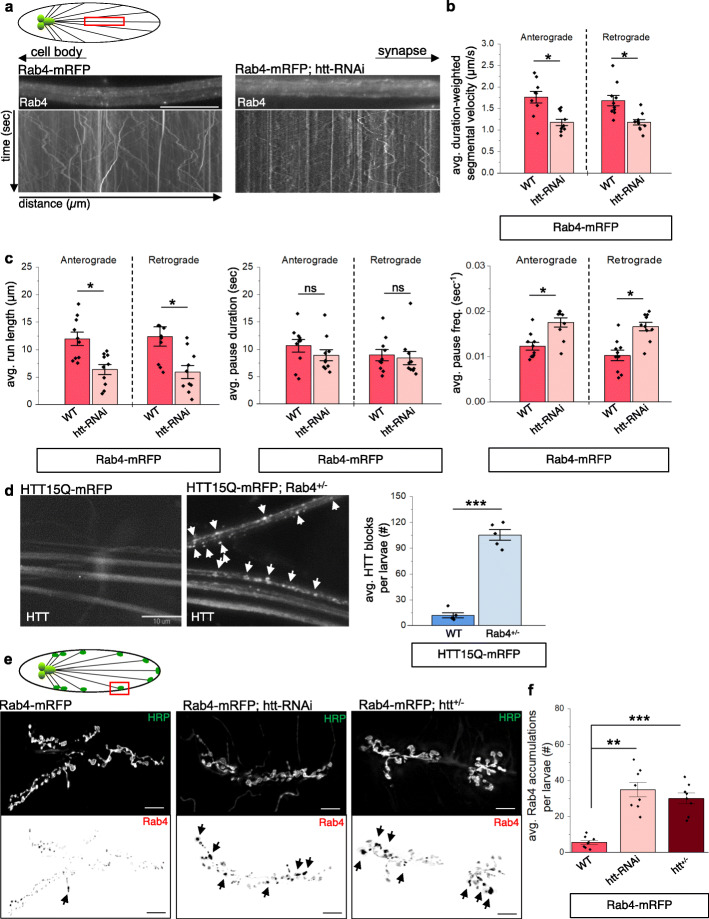
Reduction of htt disrupts the axonal motility of Rab4-vesicles. **a** Schematic diagram of larval nervous system showing the brain and segmental nerves. Red box = imaged area (90 μm). Arrows depict location of the cell bodies and synapses. Representative images from movies and corresponding kymographs from larvae expressing Rab4-mRFP or Rab4-mRFP with 70% reduction of htt (htt-RNAi). X axis = distance (μm), Y axis = time (s). Bar = 10 μm. **b** Quantification of duration-weighted segmental velocity (μm/s) indicate significant decreases in both anterograde (*p < 0.01*) and retrograde (*p < 0.01*) Rab4-mRFP vesicle velocities with 70% reduction of htt. *n* = 10, > 500 vesicles. **c** Quantification analysis shows that Rab4-vesicle run lengths (μm) are significantly decreased (*p < 0.01*), that Rab4-vesicle pause frequencies (sec^− 1^) are significantly increased (*p < 0.01*), but that Rab4-vesicle pause durations (sec) are not changed (ns) with 70% reduction of htt compared to Rab4-mRFP expressing larvae (WT). *n* = 10, > 500 vesicles. **d** Representative images of larval segmental nerves expressing HTT15Q-mRFP or HTT15Q-mRFP with Rab4^+/−^ (Rab4^MI10530^/+). Quantification of axonal blocks (#) reveal a significant increase (*p < 0.0001*) in axonal accumulations of HTT15Q-mRFP with a 50% genetic reduction of Rab4. *n* = 5. Bar = 10um. **e** Schematic diagram of third instar larval nervous system showing brain, segmental nerves, and neuromuscular junctions (NMJs). Representative images of Rab4-mRFP expressing larvae stained with HRP-FITC showing NMJs within muscle 6/7 segment A4–5 of third instar larvae from htt-RNAi (70% reduction) and htt^+/−^ (50% reduction). Top panels = HRP-FITC staining. Bottom Panel = inverted image of Rab4-mRFP fluorescence. Bar = 5 μm. **f** Quantification of the number (#) of Rab4 accumulations greater than 2μm^2^ (the average size of boutons in WT, black arrow heads) show significant increases in Rab4-mRFP accumulations with 70% (*p < 0.001*) or 50% (*p < 0.0001*) htt reduction. *n* = 8. Statistical analysis was determined using the two-sample two-tailed Student’s t-test. ns = p > 0.01, *p < 0.01, **p < 0.001, ***p < 0.0001

To test the hypothesis that HTT is involved in the axonal motility of Rab4, we generated *Drosophila* larvae co-expressing fluorescently-tagged Rab4 (Rab4-mRFP or YFP-Rab4) and htt-RNAi using the pan-neuronal Appl-GAL4 driver. We previously showed that expression of htt-RNAi with Appl-GAL4 decreased the expression of endogenous *Drosophila* htt by 70% via qPCR analysis and resulted in axonal blockages [24], while ubiquitous htt knockdown caused embryonic lethality [24]. Larvae expressing Rab4-mRFP or YFP-Rab4 alone showed robust bi-directional motility of Rab4 within the segmental nerves at velocity rates consistent with fast axonal transport (Fig.1a, S1a). Further, we note that just as expression of GFP alone did not affect axonal transport [24], tagging the N or C-terminus of Rab4 with mRFP or YFP also did not influence the bi-directional motility of Rab4 (Fig.1a, S1a). We also found that ~ 89% of Rab4-mRFP puncta co-localized with endogenous Rab4 identified using a Rab4 antibody whose epitope sequence is conserved in *Drosophila* (Fig. S1e), indicating that tagged Rab4 is likely present in the same vesicles as endogenous Rab4. Strikingly, reduction of endogenous htt disrupted the bi-directional motility of Rab4-mRFP (Fig. 1a) and YFP-Rab4 (Fig. S1a) within larval axons co-expressing Rab4-mRFP or YFP-Rab4 with htt-RNAi. Analysis of over 500 vesicle trajectories using our published custom particle tracking program [25, 59] revealed significant decreases in both the anterograde and retrograde velocities of Rab4-mRFP and YFP-Rab4 (Fig. 1b, S1b). Further, the bi-directional velocity decreases were correlated with decreases in vesicle run lengths and increases in pause frequencies (Fig. 1c, S1c). Similar observations were also seen with the htt knockout mutant (KO, Df(98E2); CG999075) [81], which exhibited Rab4-mRFP axonal blockages (Fig. S1d). Conversely, larvae expressing HTT15Q-mRFP with 50% genetic reduction of Rab4 (Rab4^MI10530^/+) resulted in HTT15Q-mRFP axonal blockages (Fig. 1d). These observations indicate that HTT and Rab4 are functionally linked during axonal transport and that HTT likely aids in the axonal motility of Rab4-containing vesicles.

To test the prediction that HTT-mediated Rab4 axonal transport defects affect the distribution of Rab4 to synapses, we next examined the *Drosophila* NMJs from larvae expressing Rab4-mRFP alone and in the context of htt depletion using htt-RNAi or heterozygous KO of htt (htt-/+). Since *Drosophila* NMJs undergo rapid structural and functional changes during larval growth [83], type-1 synaptic boutons between muscles 6/7 at larval abdominal segments A4-A5 of third instar larval were examined. Similar to previous studies [10], htt reduction did not dramatically alter the coordinated growth of NMJs (Fig. S2a). Under normal conditions, Rab4 is homogeneously distributed within NMJs (Fig. 1e). However, decreasing htt levels (either by RNAi or KO) increased in the intensity of Rab4-mRFP fluorescence at NMJs (Fig. S2b) and caused Rab4 accumulations at synaptic boutons (Fig. 1e). The area of these Rab4 accumulations were significantly greater than the average area of a synaptic bouton (2μm^2^) observed under normal conditions (Fig. 1e). These Rab4 accumulations at NMJs could result due to the capture of Rab4-containing vesicles at synapses or due to a decrease in the retrograde motility of Rab4. Indeed, previous work showed that htt-KO elevated the presence of neuropeptide at synaptic boutons [10] and synaptic accumulations were also observed in larvae expressing a point mutant in the kinesin heavy chain gene which causes autosomal dominant Hereditary Spastic Paraplegia (KHC^N262S^) [20]. Therefore, reduction of htt disrupts the axonal motility of Rab4, causing axonal and synaptic Rab4 accumulations.

### The putative moving HTT-Rab4 vesicle contains synaptic proteins, and is distinct from a putative HTT-Rab3 vesicle

**Fig. 2:**
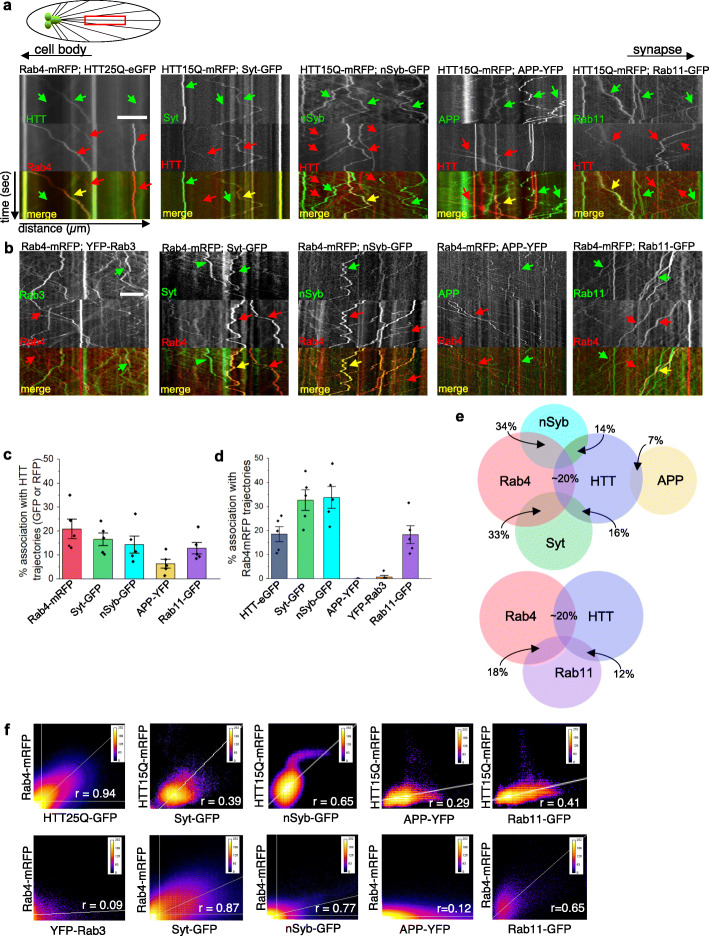
HTT moves within discrete Rab4-containing vesicles, which also contain Syt, Syb and Rab11. **a** Representative kymographs (x-axis = distance (μm) || y-axis = time(s)) from simultaneous dual-color movies from larvae expressing both HTT25Q-eGFP (green) and Rab4-mRFP (red). Co-migrating tracks that contain HTT and Rab4 are seen (yellow arrow). Note that green or red only tracks are also seen (arrowheads). Representative kymographs from larvae expressing HTT15Q-mRFP (red) and either Syt-GFP, nSyb-GFP, APP-YFP or Rab11-GFP (green). Yellow trajectories show overlaid red and green tracks. Scale = 5 μm. **b** Representative kymographs from larvae expressing Rab4-mRFP (red) and either Syt-GFP, nSyb-GFP, APP-YFP, YFP-Rab3 or Rab11-GFP (green). Yellow trajectories show overlaid red and green tracks. Scale = 5 μm. **c** Quantification of the percentage (%) of trajectories that associate with HTT trajectories (either GFP or RFP) show that ~ 21% of Rab4-mRFP trajectories overlay with HTT25Q-eGFP tracks while ~ 8% of APP-YFP trajectories overlay with HTT15Q-mRFP tracks. *n* = 5. **d** Quantification of the percentage (%) of trajectories that associate with Rab4-mRFP trajectories show that ~ 33% of Syt-GFP trajectories overlay with Rab4-mRFP tracks while 0% of APP-YFP trajectories overlay with Rab4mRFP tracks. *n* = 5. **e** Proportional Venn diagram (generated using OriginLab & RStudio) display the percent of trajectories that associate between either Rab4, HTT, nSyb, Syt or APP trajectories relative to the total number of trajectories quantified (top). An additional proportional Venn diagram displays the percent of trajectories that associate between either Rab4, HTT, or Rab11 (bottom). **f** Co-localization pixel-heat maps (ImageJ.Coloc2) generated for each of the representative movies from panel A and panel B. x-axis = GFP/YFP channel. Y-axis = RFP channel. A line near 45 degrees with a higher Pearson’s correlation coefficients R-value near 1.0 corresponds to co-localization

Although immunoprecipitation and yeast-two hybrid analysis has identified many binding partners for HTT [22, 26, 82], we were particularly interested in isolating HTT associations during its motility within axons under physiological conditions. In this context, using simultaneous dual-view imaging we tested the proposal that HTT is present within unique vesicles in vivo during its axonal motility to/from synapses within larval axons. For this, we examined larvae co-expressing HTT25Q-eGFP and Rab4-mRFP and found co-migrating (yellow trajectories) HTT- and Rab4-containing puncta (Fig. 2a, Movie S1). Quantification of merged kymographs showed that 21% of Rab4-mRFP trajectories associate with HTT25Q-eGFP trajectories, while 19% of HTT25Q-eGFP associate with Rab4-mRFP (Fig. 2c, d). Coloc2 analysis indicated a Pearson’s Correlation co-efficient of *r =* 0.94 with a 45-degree line suggesting a high degree of co-localization (Fig. 2f). In contrast, co-expression of Rab4-mRFP and YFP-tagged amyloid precursor protein (APP-YFP) did not show co-migrating APP and Rab4-containing vesicles (Fig. 2b, Movie S2). Less than 1% of APP-YFP associated with Rab4-mRFP (Fig. 2d, e). Coloc2 analysis indicated a near-horizontal line and a correlation co-efficient of *r =* 0.12 (Fig. 2f). In contrast, 7% of APP-YFP trajectories were associated with HTT15Q-mRFP trajectories with a correlation co-efficient of *r* = 0.29 (Fig. 2a, c, e, f, Movie S3), suggesting that while Rab4 and APP are likely not moving together on the same vesicle, APP can be present on a sub-population of moving HTT-containing puncta, which is likely independent from a HTT-Rab4 vesicle population.

While the native function of Rab4 remains ambiguous, there is evidence to suggest that Rab4 has a role in endosomal trafficking [49]. Further, Rab4 and HTT are both enriched at synapses [80] and both have been proposed to function during synaptogenesis [14]. To further isolate the putative moving HTT-Rab4 vesicle compartment, we next visualized larval axons co-expressing Rab4-mRFP or HTT-mRFP with candidate synaptic proteins (synaptobrevin = nSyb-GFP, synaptotagmin = Syt-GFP). Larval axons revealed co-migrating (yellow trajectories) of Rab4-mRFP and nSyb-GFP or Syt-GFP-containing puncta (Fig. 2b. Movie S4, S5). Quantification of merged kymographs showed that 34% of Syt-GFP trajectories (*r* = 0.87) and 33% of Syt-GFP trajectories (*r* = 0.77) associate with Rab4-mRFP trajectories (Fig. 2d). Additionally, HTT also co-migrated with Syb-GFP and Syt-GFP, with 14% of nSyb-GFP trajectories (*r* = 0.65) and 16% of Syt-GFP trajectories (*r* = 0.39) associating with HTT15Q-mRFP trajectories (Fig. 2c-f, Movies S6, S7). Furthermore, 12% of Rab11-GFP (a recycling endosome marker, whose motility we previously found was affected by HTT [57]) trajectories associated with HTT-mRFP (*r* = 0.41, Movie S8), while 18% of Rab11-GFP associated with Rab4-mRFP trajectories (*r* = 0.65) (Fig. 2c,d,f, Movie S9). Only < 1% of YFP-Rab3 (a synaptic Rab whose bi-directional movement we previously found was affected by HTT [77]) associated with Rab4mRFP (*r* = 0.09, Fig.2d, Movie S10). Taken together, as depicted in the proportional Venn diagrams (Fig. 2e), we propose that under physiological conditions a subpopulation of moving Rab4 vesicles likely contain HTT, Syb, Syt and Rab11 (Fig. 2e). This putative, moving HTT-Rab4 vesicle population is likely distinct from a putative HTT-Rab3 vesicle [77], and the previously identified putative APP-Rab3 vesicle [72].

### The putative moving HTT-Rab4 vesicle uses kinesin-1 and dynein motors, and the accessory protein HIP1 but not HAP1

**Fig. 3:**
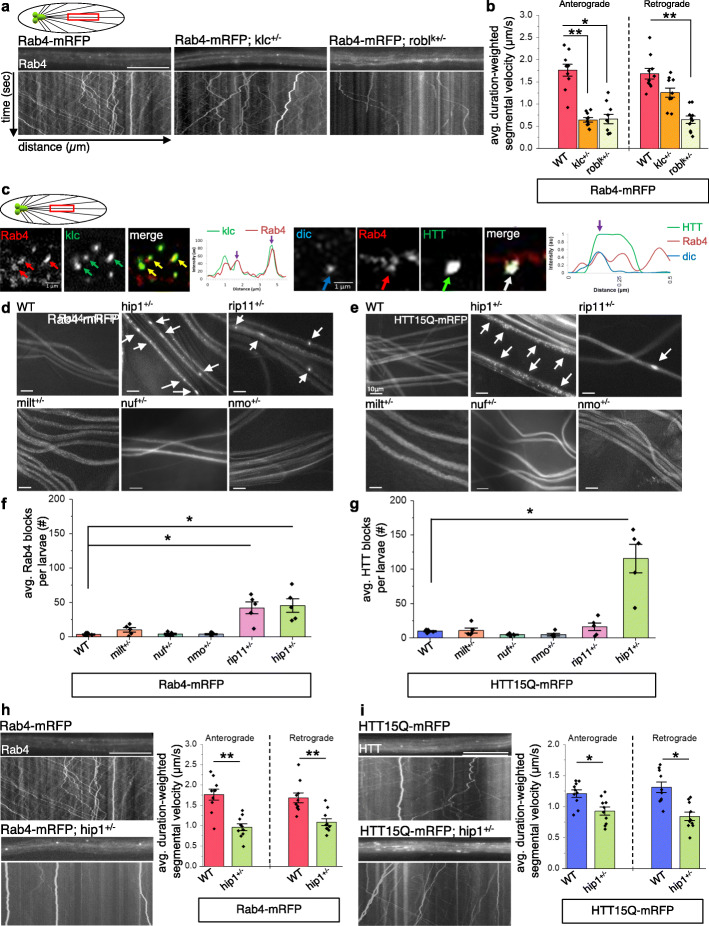
Kinesin-1, dynein, and hip1 aid the motility of HTT-Rab4 in axons. **a** Representative movies with kymographs from Rab4-mRFP larvae alone or in the context of 50% reduction of klc^8ex94^−/+ or robl^K^−/+. Bar = 10 μm. **b** Quantification of duration weighted segmental velocity (μm/s) indicates significant decreases in the anterograde Rab4-mRFP velocities with 50% reduction of kinesin (*p < 0.001*) or dynein (*p < 0.01*). 50% reduction of dynein significantly decrease retrograde Rab4-mRFP velocities (*p < 0.001)*, 50% reduction of kinesin did not (ns). *n* = 10. > 500 vesicles. **c** Sub-pixel high-resolution immunofluorescence analysis show Rab4-mRFP (red arrows) and klc (green arrows) co-localize (yellow arrows) within larval segmental nerves. Intensity-pixel plots show overlapping (purple arrows) peak intensities for both Rab4 (red) and klc (green). Rab4-mRFP (red arrows), HTT25Q-eGFP (green arrows) and DIC (blue arrows) also co-localize (white arrows). Intensity-pixel plots show overlapping (purple arrows) peak intensities for Rab4 (red) HTT (green) and DIC (blue). X axis = distance (μm), Y axis = intensity (au). Bar = 1 μm. n = 5. **d, e** Representative images of Rab4-mRFP or HTT15QmRFP larval nerves alone or in the context of 50% reduction of hip1, rip11, milt, nuf or nmo. Bar = 10 μm. **f** Quantification of the number (#) of Rab4 blocks per larvae reveal that larvae expressing Rab4-mRFP in the context of 50% reduction of rip11 (*p < 0.01*) or hip1 (*p < 0.01*) show Rab4-mRFP blocks. n = 5. **g** Quantification of the number (#) of blocks per larvae reveal that larvae expressing HTT15Q-mRFP in the context of 50% reduction of hip1 (*p < 0.01*) show HTT15Q-mRFP blocks. *n* = 5. Statistical significance was determined using the two-sample two-tailed Student’s t-test. **h** Representative movies with the corresponding kymographs from larvae expressing Rab4-mRFP alone or in the context of 50% reduction of hip1. Quantification of duration weighted segmental velocity (μm/s) indicates significant decreases in anterograde Rab4-mRFP velocities with 50% reduction of hip1 (*p < 0.001*) and in retrograde Rab4-mRFP velocities (*p < 0.001*). *n* = 10, > 500 vesicles. **i** Representative movies with the corresponding kymographs from larvae expressing HTT15Q-mRFP alone or in the context of 50% reduction of hip1. Quantification of duration weighted segmental velocity (μm/s) indicates significant decreases in anterograde velocities with 50% reduction of hip1 (*p < 0.01*) and significant decreases in retrograde HTT15Q-mRFP velocities (*p < 0.01*). *n* = 10. > 500 vesicles. Statistical significance was determined using the two-sample two-tailed Student’s t-test. ns = p > 0.01, *p < 0.01, **p < 0.001

While the anterograde motility of Rab4 within cholinergic neurons was previously shown to be primarily driven by kinesin-2 [14], work by us and others showed that the axonal motility of HTT was mediated by kinesin-1 and dynein [12, 13, 24, 49]. To test the prediction that the putative HTT-Rab4 vesicle also uses kinesin-1 and dynein motors for bi-directional motility within axons, we examined the in vivo motility behaviors of Rab4-mRFP with a 50% genetic reduction of kinesin-1 or dynein. Larvae expressing Rab4-mRFP in the context of 50% reduction of kinesin light chain (KLC, klc^8ex94^), a subunit of kinesin-1, showed significant decreases in anterograde velocities of Rab4-mRFP vesicles (Fig. 3a,b) and Rab4 accumulations (Fig. S3a). 50% reduction of dynein intermediate chain (DIC, robl^K^) significantly decreased both retrograde and anterograde velocities of Rab4-mRFP vesicles (Fig. 3a,b). While these observations are similar to what was previously seen for APP [25, 59], our analysis also suggests that kinesin-1 and dynein motor activities on Rab4 vesicles are likely functionally coupled during axonal transport. We also found that larvae expressing Rab4-mRFP in the context of 50% reduction of either kinesin-2 (klp64d) or kinesin-3 (unc-104) caused Rab4 accumulations (Fig. S3a), indicating that the motility of Rab4 is likely influenced by all 3 kinesin motors. However, we propose that since the putative motile HTT-Rab4 vesicle complex also contained synaptotagmin, our moving HTT-Rab4 vesicle is likely distinct from the kinesin 2-mediated Rab4 vesicles isolated in cholinergic neurons, which lacked synaptotagmin [14]. Further, immunofluorescence imaging of larval axons showed that Rab4-containing puncta co-localize with KLC and DIC (Fig. 3b). HTT is also present with Rab4 and KLC or DIC containing puncta (Fig. 3c). These findings, together with our previously published work on HTT and molecular motors [24, 34], indicate that the putative HTT-Rab4 vesicle is distinct from other Rab4 vesicles and uses both kinesin-1 and dynein for its bi-directional movement within axons.

Previous work showed that huntingtin-associated protein 1 (HAP1) functions as an accessory protein to mediate associations between HTT-containing vesicles and motors in mammals [16, 50]. Similar to HTT, HAP1 was also shown to move both anterogradely and retrogradely within axons [7]. To test the proposal that associations between molecular motors and the putative HTT-Rab4 containing vesicle are mediated by accessory proteins, we undertook a candidate approach to isolate potential proteins involved in the motility of the putative HTT-Rab4 vesicle in vivo. HTT moves bi-directionally within larval axons in vivo (Fig. S4a) [7, 36, 75], although *Drosophila* lacks a true homologue of HAP1. Reduction of endogenous *Drosophila* htt (using htt-RNAi or the knock-out mutant) disrupted the motility of non-pathogenic human HTT indicating that the motility of HTT15Q-mRFP is dependent on endogenous *Drosophila* htt (Fig. S4a). One possibility for the HAP1 independent motility of HTT within larval axons is that the HAP1-like protein, milton, which exhibits mild homology (~ 50%) with the N-terminal domain of mammalian HAP1 [70], contributes to the motility of HTT in flies. To test this prediction, we examined larvae expressing HTT15Q-mRFP and Rab4-mRFP with 50% reduction of milton (milt^K04074^/+) and found no axonal transport defects (Fig. 3d-g), suggesting that milton does not affect the motility of the putative HTT-Rab4 vesicle and is unlikely to be a part of the putative HTT-Rab4 vesicle complex.

To test whether other huntingtin interacting proteins are involved in the motility of the putative HTT-Rab4 vesicle we tested a potential candidate; huntingtin-interacting protein 1 (HIP1), which participates in clathrin-mediated endocytosis [38, 51] and has been implicated in endosomal trafficking [31]. HIP1 also associates with membrane-bound HTT but exhibits decreased affinity for HTT with increasing polyQ tracks [51]. Larvae expressing HTT15Q-mRFP or Rab4-mRFP with 50% reduction of *Drosophila* HIP1 (hip1^MI05905^/+) caused HTT/Rab4 axonal blockages (Fig. 3d-g) and decreased both the anterograde and retrograde motility of either HTT15Q-mRFP or Rab4-mRFP vesicles (Fig. 3h-i). These motility defects were concomitant with decreases in vesicle run lengths (Fig. S4b, c). These observations indicate that HIP1 contributes to the in vivo motility of the putative HTT-Rab4 vesicle within larval axons.

Several Rab-associated effector proteins are known to regulate the delivery of Rabs to specific intracellular locations [11, 13, 56, 79]. Previous work showed that optineurin binds to Rab8 to link myosin and HTT for motility on actin filaments [63]. However, reduction of the *Drosophila* homolog of optineurin, nemo (nemo^P1^/+), did not affect the axonal motility of HTT15Q-mRFP or Rab4-mRFP (Fig. 3d-g). Further, reduction of nuf (nuf^MB09772^/+), which binds Rab11 to influence recycling endosome organization in a dynein-dependent manner [61], also did not affect the axonal motility of HTT or Rab4 (Fig. 3d-g). However, reduction of rip11 (rip11^KG02485^/+), a Rab11 effector thought to regulate endosomal trafficking via kinesin associations [66], disrupted the axonal motility of Rab4 causing a significant amount of Rab4-mRFP containing axonal blockages (Fig. 3d,f), while no significant affects were seen with HTT15Q-mRFP (Fig. 3e, g). Simultaneous dual-view imaging revealed that Rab4-mRFP and rip11-GFP co-localize within larval axons (Fig. S3b). Taken together, our observations suggest that at least one Rab-associated effector protein, rip11 is likely involved in the axonal motility of Rab4 vesicles, but not for the axonal motility of the putative HTT-Rab4 vesicle.

**Fig. 4:**
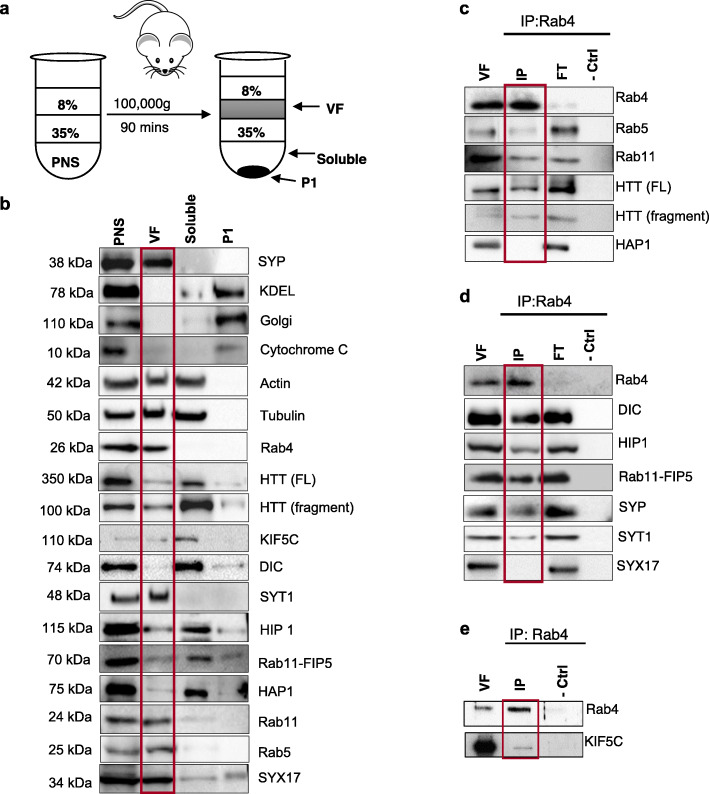
HTT, HIP1, Rab11-FIP5 and motors associate with isolated Rab4-vesicle membranes. **a** Schematic diagram of mouse brain homogenate fractionation into perinuclear supernatant (PNS), vesicle fraction (VF), soluble, and heavy membrane (P1) fractions by ultra-centrifugation and sucrose gradient separation. **b** Representative western blot of each fraction probed with antibodies to synaptotagmin-1 (SYT1, VF), synaptophysin (SYP), KDEL (ER), Golgi, Cytochrome C (mitochondria), Actin and Tubulin (loading controls), Rab4, HTT, KIF5C (kinesin), DIC (dynein), HIP1, Rab11-FIP5, HAP1, Rab11, Rab5 or Syntaxin17 (SYX17). Rab4, SYT1, SYP, SYX17, Rab5, and Rab11 are enriched in the VF, while HTT, HIP1, HAP1, Rab11-FIP5, KIF5C and DIC are present in the VF and the soluble fraction. KDEL, Golgi and cytochrome C are enriched in P1. **c** Representative western blot of an immunoprecipitation of Rab4-containing VF, probed with HTT, Rab5, Rab11, and HAP1. HTT and Rab11 show their presence in the Rab4 IP, but HAP1 is not seen. No bands are seen in the negative no antibody control (−Ctrl). **d** Representative western blot of an immunoprecipitation of Rab4-containing VF, probed with DIC, HIP1, Rab11-FIP5, SYP, SYT1, and SYX17. DIC, HIP1, Rab11-FIP5, SYP, and SYT1 are all present in the Rab4 IP, but not SYX17. No bands are seen in the negative no antibody control (−Ctrl). **e** Representative western blot of an immunoprecipitation of Rab4-containing VF, probed with KIF5C. KIF5C show that these are present in the Rab4 IP. *n* = 3

Next, we biochemically probed the existence of a putative HTT-Rab4 vesicle by first isolating Rab4-containing vesicle membranes from mouse brains. Mouse brains were fractionated and the post-nuclear supernatant (PNS), vesicle fraction (VF), soluble, and heavy-membrane fractions (P1) were isolated as previously done [15] (Fig. 4a). Western blot analysis indicated that full-length HTT (~350kD), Rab4, kinesin-1 (KIF5C) and dynein (DIC) were all present in the VF containing synaptotagmin-1 (SYT1) (Fig. 4b). HIP1, Rab11-FIP5 (the mammalian homolog of rip11), HAP1 and Rab11 were also seen in the VF (Fig. 4b). Note that Rab4 is enriched in the VF with very little seen in the soluble fraction. We next isolated Rab4-containing membranes by immunoprecipitating (IP) Rab4 from the VF (Fig. 4c-e). Western blot analysis of the Rab4-membrane IP showed both the full length and N-terminal HTT fragments in the Rab4 membrane IP (Fig. 4c). Kinesin-1 (KIF5C) and dynein (DIC) motors were also present in the Rab4 membrane IP (Fig. 4d, e). Intriguingly, Rab11, Rab11-FIP5 and HIP1 were also seen in the Rab4-membrane IP, while HAP1 was not (Fig. 4 c,d). Note that synaptotagmin (SYT1) and synaptophysin (SYP) were also pulled downed in the Rab4-membrane IPs, while syntaxin-17 (SYX17, an autophagy-related SNARE protein [33]) was not (Fig. 4d). Taking our genetic and biochemical observations together, our results indicate the existence of a putative HTT-Rab4 vesicle-motor complex containing HIP1, but not milton/HAP1, rip11, nemo, or nuf in fly and mammalian neurons (Fig. S9a).

### Rab4 motility is disrupted in HD neurons due to aberrant associations with motors and accessory proteins

**Fig. 5:**
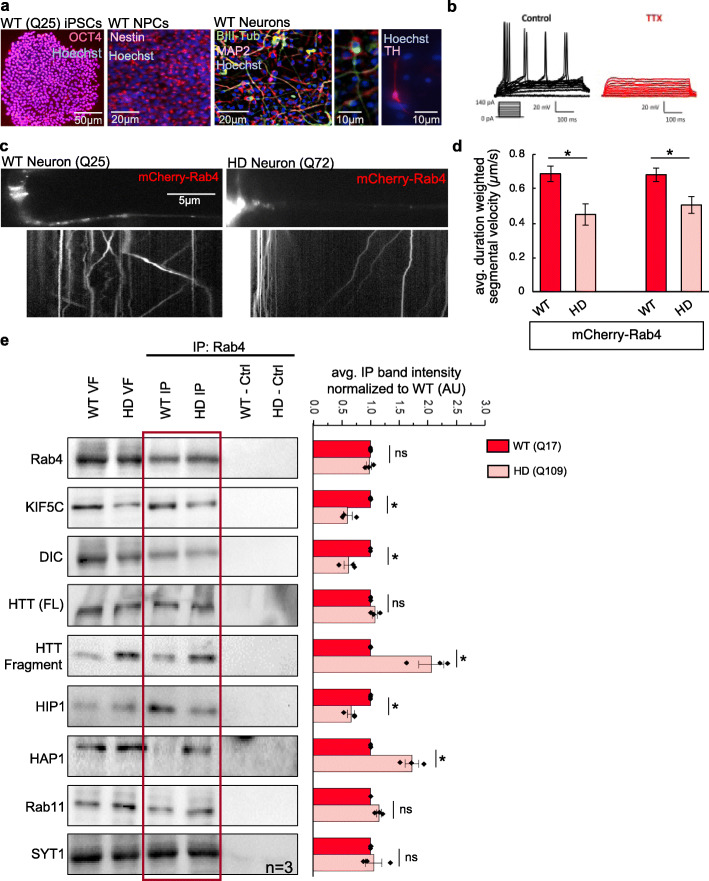
The axonal motility of Rab4 is disrupted in human HD iNeurons. **a** Representative images from normal (WT, Q25) human iPSCs stained with the pluripotent marker OCT4, the neuronal precursor (NPC) marker Nestin and the mature neuronal markers MAP2 and βIII-Tubulin. Hoechst stains nuclei. Differentiated neurons show Tyrosine hydroxylase (TH) positive staining. Bar = 50, 20, or 10 μm. **b** Electrophysiological analysis of normal human neurons differentiated from iPSCs show action potentials which are abolished in the presence of TTX. **c** Representative movies with the corresponding kymographs from normal (WT, Q25) and HD (Q72) neurons transfected with mCherry-Rab4. Bi-directional Rab4 motility is observed in WT neurons. HD neurons show few moving mCherry-Rab4 puncta. **d** Quantification of duration weighted-segmental velocity (μm/s) revealed that both the anterograde and retrograde global velocities of mCherry-Rab4 puncta are significantly decreased (*p < 0.01, p < 0.01*) in HD neurons compared to WT neurons. *n* = 7 independent neurites per genotype from 2 cultures. ns = p > 0.01, *p < 0.01. Statistical significance was determined using the non-parametric Wilcoxon–Mann–Whitney rank sum test. **e** Representative western blot of a Rab4-containing VF IP from WT (Q17) or HD (Q109) neurons that was probed for Rab4, KIF5C, DIC, HTT, HIP1, HAP1, Rab11, and SYT1. The HTT fragment, HAP1, and Rab11 bands appear darker in HD IP compared to WT IP fraction. Conversely, the HIP1, KIF5C and DIC bands appear lighter in the HD IP compared to WT IP fraction. No bands are seen in the negative no antibody control (−Ctrl). Band intensity ratios of the IP fraction were analyzed for each probe from WT and HD neurons and normalized to the WT IP band intensity for that probe (AU). When comparing WT to HD, band intensity ratios showed a significant decreased for KIF5C (*p < 0.05*), DIC (*p < 0.05)*, and HIP1 (*p < 0.05)*, while significant increases were seen for the HTT fragment (*p < 0.05*) and HAP1 (*p < 0.05*). *n* = 3. ns = p > 0.05, *p < 0.05. Statistical significance was determined using the two-sample two-tailed Student’s t-test

We next tested whether the motility of the putative HTT-Rab4 vesicle is disrupted in HD. In this context, we first terminally differentiated neurons derived from iPSCs from age and sex matched normal (Q25, Q17) and HD (Q72, Q109) individuals. Using previously established protocols, OCT-4+ iPSCs were first differentiated into Nestin+ neuronal precursor cells (NPCs), and then terminally differentiated into neurons (β-III Tubulin+ and MAP2+ iNeurons) (Fig. 5a, S5a). Electrophysiology indicated the presence of functional iNeurons (Fig. 5b, S5b) capable of eliciting action potentials. Na + or K+ currents were inhibited by TTX or TEA demonstrating the presence of functional Na + and K+ channels in both WT and HD iNeurons with no defects detected at this stage (Fig. 5b, S5b-d). Next, we transfected WT and HD iNeurons with mCherry-Rab4a-7 and observed the motility dynamics of mCherry-Rab4-vesicles in iNeurons using the same protocol used for imaging Rab4-mRFP-vesicle motility in *Drosophila* larval axons (Figs. 1, 3). Similar to what was observed in larval axons, Rab4 moves bi-directionally within WT iNeurons (Fig. 5c, Movie S11) with the anterograde and retrograde Rab4-vesicle velocities (Fig. 5d) and run lengths (Fig. S5e) comparable to our observations in larval axons (Fig. 1b-c, S1b-c). In contrast, the bi-directional motility of mCherry-Rab4-vesicles was significantly perturbed in HD iNeurons (Fig. 5d, Movie S12). Concomitant with decreases in mCherry-Rab4-vesicle velocities, significant decreases in both the anterograde and retrograde vesicle run lengths were observed (Fig. S5e).

One possible mechanism for how expanded polyQ-HTT could disrupt the axonal motility of Rab4-vesicles is by disrupting the association of Rab4-vesicles with motors and/or accessory proteins which regulate motor attachment. Indeed, previous work showed that polyQ-HTT disrupted the association of HAP1 with p150glued [21] and KLC [62], perturbing the axonal transport of BDNF. Moreover, polyQ-HTT also decreased HTT’s affinity for HIP1 [51], while its affinity for HAP1 was increased [41]. To test the proposal that defects in Rab4 motility in HD iNeurons is due to the disruption of associations between Rab4-vesicles with motors and/or accessory proteins, we first isolated Rab4-containing membranes from HD and WT iNeurons. Western blot analysis of the SYT1 positive membrane fraction from WT and HD iNeurons showed full-length (FL) and N-terminal fragments of HTT, Rab4, Rab11, KIF5C, dynein, HIP1, HAP1 and Rab11FIP5 (Fig. 5e). We next immunoprecipitated the Rab4-containing VF from both WT and HD iNeurons. Co-IP analysis of HD VFs revealed altered associations between Rab4 and known transport regulatory proteins compared to WT VFs (Fig. 5e). HD iNeurons exhibited significant reductions in the level of motor protein subunits (KIF5C and DIC) and HIP1 that immunoprecipitated with Rab4 suggesting decreased associations between vesicular Rab4 in HD iNeurons compared to WT iNeurons (Fig. 5e). In contrast, increased levels of HAP1 was observed in Rab4-membranes from HD iNeurons (Fig. 5e), suggesting that HAP1 exhibits greater binding affinity for expanded polyQ-HTT compared to WT iNeurons, perhaps due to aberrant associations between HAP1 and the putative polyQ-HTT-Rab4 vesicle similar to what has been previously reported [21, 41, 51, 62]. Additionally, while changes to the total level of proteins were seen between WT and HD neurons (Fig. S7), we also found that HD iNeurons had increased levels of HTT N-terminal fragment pulled down with Rab4-membranes compared to WT iNeurons, indicating that perhaps cleaved polyQ-HTT fragments associate more strongly with Rab4-containing vesicles (Fig. 5e). Immunofluorescence analysis showed that Rab4 and HTT co-localize in WT iNeurons, while Rab4 and HTT were present in axonal accumulations in HD iNeurons (Fig. S6a, b). High resolution imaging showed that Rab4 puncta also co-localize with DIC and HIP1 in WT iNeurons, but this co-localization was disrupted in HD iNeurons (Fig. S6c, d). Taken together, our observations indicate potentially significant disruption of the putative polyQ-HTT-Rab4 vesicle complex and molecular motors/accessory proteins under HD disease conditions, which likely contributes to the disruption of the axonal motility of polyQ-HTT-Rab4-containing vesicles (Fig. S9b).

### Synaptic dysfunction and behavioral defects caused by pathogenic HTT are rescued by excess Rab4

**Fig. 6:**
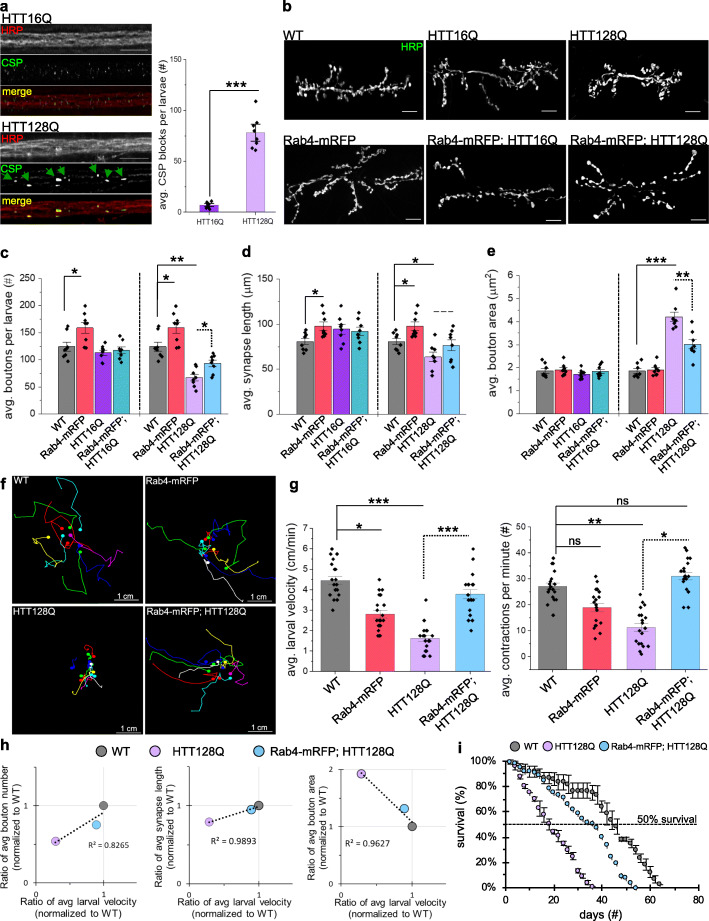
Expression of Rab4-mRFP rescues synaptic deformation and larval crawling defects caused by pathogenic HTT**. a** Representative images from larvae expressing HTT16Q or HTT128Q immunostained for the synaptic marker CSP and the neuronal membrane marker HRP-TxRED. Smooth staining is seen in HTT16Q larval nerves with the synaptic vesicle marker, CSP. Larval nerves expressing HTT128Q show axonal accumulations of CSP. Quantification of avg. CSP blocks per larvae (#) show a significant number of axonal blocks in larvae expressing HTT128Q compared to larvae expressing HTT16Q (*p < 0.0001*). *n* = 8. **b** Representative images of NMJs from muscle 6/7 segment A4–5 of WT larvae, larvae expressing HTT16Q or HTT128Q, or larvae co-expressing either HTT16Q or HTT128Q with Rab4-mRFP that have been stained with HRP-FITC. Note, NMJs of HTT16Q expressing larvae look similar to NMJs of WT larvae. **c** Quantification of the avg. number of boutons (#) per larvae revealed a significant decrease in the number of boutons at neuromuscular junctions of HTT128Q expressing larvae compared to WT (*p < 0.001*). Co-expression of Rab4-mRFP with HTT128Q rescued the number of boutons (ns) compared to WT, with a significant increase (*p < 0.01*) compared to HTT128Q larvae alone. *n* = 8. **d** Quantification of the avg. synapse length (μm) per larvae revealed a significant decrease in synapse length at neuromuscular junctions of HTT128Q expressing larvae compared to WT (*p < 0.001*). Co-expression of Rab4-mRFP with HTT128Q rescued the number of boutons (ns) compared to WT. *n* = 8. **e** Quantification of the avg. bouton area (μm^2^) per larvae revealed a significant increase in bouton area at neuromuscular junctions of HTT128Q expressing larvae compared to WT (*p < 0.0001*). Co-expression of Rab4-mRFP with HTT128Q rescued the number of boutons (ns) compared to WT, which were significant decreased in area compared to HTT128Q expressing larvae (*p < 0.001*). *n* = 8. **f** WT larvae, larvae expressing Rab4-mRFP or HTT128Q, and larvae co-expressing Rab4-mRFP with HTT128Q were subjected to larval crawling assays for 1.5 mins at 25C, 60% humidity. **g** Quantification of larval crawling behaviors (larval velocity = cm/min & larval contractions = contractions/minute) show significant decreases in both larval velocity (*p < 0.0001*) and contractions (*p < 0.001*) in HTT128Q larvae. Larvae co-expressing Rab4-mRFP and HTT128Q show no changes in crawling velocity (ns) or contractions (ns) compared to WT. *n* = 20. **h** Correlation scatterplots for ratios of avg. larval velocity normalized to WT (from panel **g**) compared to ratios of either bouton number (from panel **c**), synapse length (from panel **d**), or bouton area (from panel **e**) normalized to WT. Corresponding R-values from linear regressions indicate a positive correlation between crawling velocities and synaptic dysfunction. **i** Survival of HTT128Q and WT adult flies were measured for 70 days at 25 °C with 60% humidity. Quantification of survival percentage (%) reveals that HTT128Q flies do not survive beyond 40 days, with 50% not surviving beyond 20 days, while WT flies survived until day 63, with 50% of the WT flies surviving beyond 40 days. Adult flies co-expressing HTT128Q and Rab4-mRFP show a partial rescue of survival until day 53, with 50% flies surviving to day 40. *n* = 3 independent experiments, total of 60 adult flies per genotype. ns = p > 0.01, *p < 0.01, **p < 0.001, ***p < 0.0001. Statistical significance was determined using the two-sample two-tailed Student’s t-test

We previously showed that disruption of axonal transport via loss of motor function caused synaptic defects [34] and larval locomotor defects which were correlated with the number of axonal blockages [2]. To test whether pathogenic polyQ-HTT also disrupts axonal transport and causes synaptic and behavioral defects in vivo, we examined larvae expressing pathogenic polyQ-HTT. Larvae expressing HTT128Q exhibited axonal blockages with the synaptic vesicle marker CSP (cysteine string protein), in contrast to larvae expressing non-pathogenic HTT16Q (Fig. 6a). These CSP blockages also contained Rab4 and HTT (Fig. S8a, b), similar to what was observed in HD iNeurons (Fig. S6a, b). We next tested the proposal that disruption of Rab4 transport mediated by pathogenic polyQ-HTT affects the coordinated growth of synapses, by examining type1 synaptic boutons in NMJs between muscle 6/7 at larval abdominal segments A4-A5 similar to our previous analysis [2, 34]. While NMJs from larvae expressing non-pathogenic HTT (HTT16Q) were comparable to WT, NMJs from larvae expressing HTT128Q showed significant changes to the coordinated growth of NMJs (Fig. 6b-e). The total number of synaptic boutons (Fig. 6c) and the average synaptic length (Fig. 6d) were significantly decreased, while the average size of synaptic boutons (Fig. 6e) was significantly increased in HTT128Q larvae compared to WT or HTT16Q larvae.

To test the proposal that synaptic defects contribute to behavioral defects, we next examined larval crawling velocities and peristaltic body wall contractions. The *Drosophila* neuronal network called the central pattern generator controls the coordination of peristaltic movement of larval body muscles, which result in crawling [19, 47, 48]. A full peristaltic contraction of the body wall initiates at the posterior of the larva and propagates in a wave-like manner to the anterior of the larva, terminating at the mouth hooks. Since larval crawling speeds increase with the frequency of contractions, speed, body muscle contractions and distance traveled are all thought to be correlated [1, 5]. Similar to motor protein mutant larvae [2], larvae expressing HTT128Q showed significantly decreased larval contractions concomitant with decreased larval velocities compared to WT larvae (Fig. 6f, g). Further analysis indicated a high Pearson’s correlation coefficient between larval crawling velocities and synaptic defects for HTT128Q larvae compared to WT larvae, suggesting a high linear association between these two variables (Fig. 6h). Moreover, while HTT128Q expressing adult flies die or do not enclose to adults at 29 °C, at 25 °C these flies showed decreased survival rates compared to adult WT flies (Fig. 6i). Taken together, these observations are strikingly similar to what was observed for loss of motor proteins [2], indicating that perturbation of axonal transport mediated by pathogenic polyQ-HTT causes dysfunction in the coordinated growth of NMJs, resulting in larval locomotion defects and adult lethality.

Previous work showed that expression of Rab11 ameliorated synaptic and behavioral dysfunction in a *Drosophila* HD model [60] and rescued neurodegeneration in HD mice [69]. Since it was suggested that proper Rab4 transport and localization is important for the regulation of coordinated synaptic organization [14], we next tested the proposal that supplying excess Rab4 can restore polyQ-HTT-mediated axonal transport defects, synaptic dysfunction and behavioral deficits, by generating larvae co-expressing Rab4-mRFP with HTT128Q. Although larvae co-expressing Rab4-mRFP with HTT128Q still contained axonal blockages, there was no significant increase in the extent of blockages when compared to HTT128Q larvae (Fig. S8a, b). However, while HTT128Q larval NMJs showed synaptic defects (Fig. 6b-e), larvae co-expressing Rab4-mRFP with HTT128Q did not and were strikingly similar to WT (Fig. 6b-e). These larval NMJs showed smaller synaptic boutons in contrast to HTT128Q larval NMJs (Fig. 6e). Increased numbers of boutons and an increased average synaptic length were also observed, compared to HTT128Q larvae (Fig. 6c-d). Strikingly, the level of Rab4 at the NMJs of larvae co-expressing Rab4-mRFP and pathogenic HTT128Q was significantly increased compared to larvae expressing Rab4-mRFP alone or with HTT16Q (Fig. S8c). Further, while larval crawling velocities and larval body wall contractions in larvae expressing pathogenic HTT128Q were significantly decreased, larvae co-expressing Rab4-mRFP and pathogenic HTT128Q were not and were strikingly comparable to WT larval crawling velocities and body wall contractions (Fig. 6f,g). Correlation analysis indicates that larvae co-expressing Rab4-mRFP and HTT128Q are comparable to WT larvae (Fig. 6h). Furthermore, the adult survival rates observed for flies expressing HTT128Q were also partially rescued in flies co-expressing Rab4-mRFP and pathogenic HTT128Q (Fig. 6i). Taken together, these observations suggest that disrupted transport of Rab4 mediated by pathogenic HTT likely causes loss of Rab4 function at the synapses, affecting larval locomotion defects, and that providing excess Rab4 restores these defects. Our observations are strikingly similar to previous work which showed that overexpression of Rab11 reversed mutant HTT-mediated synaptic dysfunction and behavioral deficits [60]. Therefore, since the putative HTT-Rab4 vesicle complex likely contains Rab11 (Figs. 2, 4, 5), it is not surprising that supplying excess Rab4 also restored the polyQ HTT-mediated synaptic defects and behavioral deficits perhaps due to Rab4-Rab11-mediated endosomal functions at NMJs.

## Discussion

Despite growing evidence that HTT plays an important role during axonal transport, the specific vesicle complexes that HTT is present on, and the cargo that HTT carries during long-distance axonal transport in vivo remains elusive. This is, in part, due to the challenge of isolating moving HTT complexes within living axons in a whole organism. Here we use *Drosophila* genetics coupled with in vivo imaging to identify a putative moving HTT-Rab4 vesicle complex containing synaptic SNARE proteins synaptotagmin and synaptobrevin, and the recycling endosome marker Rab11. While this putative HTT-Rab4 vesicle uses kinesin-1 and dynein for its bi-directional movement within axons, the HTT accessory protein, HIP1, is also required, but not HAP1. Disruptions in the axonal motility of this putative HTT-Rab4 vesicle perhaps via pathogenic polyQ-HTT caused synaptic dysfunction, locomotion defects, and reduced lifespan in *Drosophila*. Intriguingly, overexpression of Rab4 rescued these pathogenic polyQ-HTT-mediated phenotypes suggesting that HTT and Rab4 function together for the coordinated growth of synapses. Therefore, identification of a discrete HTT-Rab4-vesicle population has important implications for how defects in axonal transport contributes to HD pathology, highlighting Rab4 and the endosomal pathway as a potential novel therapeutic target for early intervention prior to neuronal loss and behavioral defects observed in HD.

### The physiological relevance of a moving axonal HTT-Rab4 vesicle complex and its disruption in HD

Our observations suggest that only a fraction of Rab4-containing vesicles contain HTT. This is not surprising since there are likely many different motile vesicles which are composed of different proteins. Indeed, Hinckelmann et al. identified several different motile vesicles that contained p50-GFP [28]. In this study, Rab4B was also one of the candidates identified with p50-GFP, although it was unclear whether this vesicle class actually moved within axons. More recently, HTT has been implicated in the transport of APP [9] which is consistent with our in vivo analysis which shows that HTT and APP co-migrate within vesicles (Fig. 2a). However, the HTT-APP co-migrating vesicle complex is not part of the putative HTT-Rab4 motile vesicle complex we isolated in this study. We previously showed that HTT is present with Rabs 2, 3, 7, 11 and 19, but not with 11 other neuronal Rabs [57, 77]. Therefore, we speculate that there are likely several different motile vesicle complexes containing either HTT or Rab4 that are moving bi-directionally within axons. Isolating these specific moving vesicle complexes and elucidating their functional significance becomes challenging.

Our investigation into the putative moving HTT-Rab4-containing vesicle demonstrates genetic and functional interactions between Rab4 and molecular motors kinesin-1 and dynein, similar to the observation that kinesin-1 and dynein move HTT in *Drosophila* and mammalian neurons [12, 50, 53]. Previous work suggested that the anterograde motility of Rab4 is mediated by kinesin-2 and kinesin-3 [14, 32]. Although these studies did not examine kinesin-1, our analyses revealed that all 3 classes of kinesin motors are likely involved in the axonal motility of Rab4 vesicles. It is probable that compartmentalized signaling in neurons is dependent on specific motor protein-mediated active transport [74], and a plethora of adaptor proteins may aid in coordinating the motility of diverse populations of motor cargo-complexes [29, 65]. Indeed, selective filtering of motor-cargo complexes has been proposed to contribute to the preferential trafficking and segregation of cellular components within axons and dendrites in polarized neurons [55, 67]. Therefore, it is likely that different types of Rab4-containing vesicle populations exist within axons/dendrites, each carrying a unique set of proteins propelled by specific kinesin motors. Alternatively, specific types of Rab4-vesicles are likely transported in certain neuronal types by particular kinesin motors. Although these two possibilities are not mutually exclusive, the putative axonal HTT-Rab4 vesicle complex we identified, which contains synaptic SNARE proteins and is transported in motor neurons by kinesin-1, is likely distinct from the kinesin-2/3-mediated Rab4 vesicles in cholinergic neurons, which lacked synaptotagmin [14]. Further, given the rates of the in vivo velocities of Rab4 and HTT that we observe, a processive kinesin motor such as kinesin-1 or a combination of kinesin-1 and -3 motors must be involved. Since reductions in kinesin-3 disrupted Rab4 motility we propose that kinesin-3 may also be involved in the motility of HTT-Rab4 vesicles we identified. Indeed, a recent study showed that kinesin-1 and -3 can function together to support fast, highly processive anterograde motility [44]. Therefore, while the HTT-Rab4 vesicle uses kinesin-1 (perhaps with kinesin − 3), this particular vesicle complex is likely distinct from the Rab4 vesicle that utilize kinesin-2 for its axonal motility. Consistent with this proposal, we found that Rab4 motility but not HTT, was disrupted by reduction of the Rab11 effector rip11/Rab11-FIP5, a binding partner for kinesin-2 [66]. Interactions between Rab4A and the cytoplasmic dynein light intermediate chain-1 have also been proposed [6]. Therefore, discrete classes of moving Rab4-containing vesicles likely exist, with unique roles in axonal and synaptic function.

Rab4 has been shown to be present on both early endosomes marked by Rab5 and recycling endosomes marked by Rab11 [68]. However, since we found that htt reduction affected the in vivo axonal motility of Rab4 and Rab11, but not Rab5 [77], and that HTT-Rab4 and Rab11 co-migrated within axons (Fig. 2), the axonal HTT-Rab4 vesicle we isolated is likely not a Rab5-containing early endosome. Consistent with this proposal, the Rab4 effector GRASP-1 can segregate Rab4 from Rab5-positive early endosomes and can coordinate Rab4 coupling to Rab11-positive recycling endosomes [30]. The Rab4/Rab11-GRASP1 compartment, which also contains the endosomal SNARE syntaxin-13 has been proposed to carry the glutamate receptor AMPAR. GRASP1, which is essential for synaptic plasticity and for spine morphology was found to regulate AMPAR recycling at the synapse [30]. The axonal HTT-Rab4 vesicle complex we isolated contains Rab11 and the SNARE proteins synaptotagmin and synaptobrevin, but not APP or Rab3 (Figs. 2, 4, 5, S9a), and likely has a unique role at the synapse. Further, while recycling of synaptic proteins are essential for the maintenance of synapse function, SNARE proteins such as synaptotagmin and synaptobrevin may also have roles during endosome trafficking [46], perhaps to recruit tethering factors [35, 37] for motor attachment. Indeed, the endosomal-associated transport regulator, HIP1, aids the motility of the HTT-Rab4 vesicle (Fig. 3h, i, S4b, c). Similar to HTT and Rab4, HIP1 is highly expressed in the brain, is membrane bound (Fig. 4), and participates in clathrin-mediated endocytosis [17, 38]. Therefore, the putative HTT-Rab4 vesicle (Fig. S9a) we isolated likely plays an important role in transporting particular neurotransmitters and/or neurotrophic factors such as BDNF [45, 78], which are essential for synaptic homeostasis. Indeed, reduction of HTT disrupted the axonal motility of Rab4 resulting in Rab4 accumulations within synaptic boutons (Fig. 1e, f). Although further study is needed to fully identify the protein composition of this unique HTT-Rab4 vesicle population and its exact role in synaptogenesis, we propose that endogenous HTT likely functions as a scaffold to link unique Rab4-containing cargo complexes to kinesin-1 and dynein to regulate their bi-directional motility within axons, which could also dictate the cell autonomous mechanisms seen in HD.

Our observations demonstrate that the moving HTT-Rab4 vesicle complex is disrupted in HD patient iNeurons (Fig. 5, S5–7). While disruption of long-range transport within axons has been linked to many neurodegenerative diseases, including HD, several potential mechanisms could exist for how the axonal motility of the putative HTT-Rab4 vesicle is disrupted. One mechanism involves the sequestering of proteins into cytoplasmic inclusions observed within neuronal projections during HD progression [52]. Indeed, motors such as dynein were isolated in cytoplasmic inclusions indicating that disruption of cargo-motor complexes could occur due the sequestering of motors into inclusions [24, 58, 75]. Further, since expansion of polyQ-HTT decreases the HTT binding affinity of HIP1 [51], while the binding affinity for HAP1 was increased [75], perhaps aberrant binding affinities mediated by polyQ expansion can disrupt associations between the HTT-Rab4 vesicle complex and motors. In fact, our observations show a decreased affinity of Rab4 for HIP1 and molecular motors in HD iNeurons (Fig. 5e). Interestingly, although HAP1 is likely not involved in the motility of HTT-Rab4 under normal conditions, under pathogenic polyQ-HTT conditions, HAP1 appears to bind more strongly to Rab4 membranes in HD iNeurons (Fig. 5e). This is consistent with previous work that showed that polyQ-HTT exhibits a greater affinity for HAP1 and disrupts the association of HAP1 with p150 [21] and KLC [62], perturbing BDNF transport and decreasing the intracellular level of TrkA. While the sequestration and binding affinity mechanisms may not be mutually exclusive, fully isolating the mechanistic steps involved in the disruption of the axonal motility of various HTT containing vesicles in HD would enable us to better identify the nature of the cell-autonomous defects seen in HD.

### Excess Rab4 ameliorates synaptic and behavioral dysfunction caused by pathogenic HTT in vivo

Studies have linked perturbation in long-distance axonal transport with synaptic dysfunction [2, 34, 52]. Our observations also shed light on the importance of the putative HTT-Rab4 vesicle population in the context of HD, where perturbation of HTT-Rab4 motility contributes to the synaptic and behavioral defects seen in HD. The fact that Rab4 expression reversed polyQ-HTT-mediated synaptic defects, larval crawling deficits, and adult lifespan (Fig. 6b-i), is strikingly complementary to previous work that showed that expression of Rab11 also rescues synaptic dysfunction and behavioral defects in flies [69] and mice [60]. These findings suggest that the synaptic and behavioral defects caused by polyQ-HTT are, at least partly due to perturbed Rab4 function in the axonal-endosomal pathway. An alternate possibility is that polyQ-HTT alters the activity of Rab4. Indeed, many neuronal processes such as the extension of axonal growth cone and the maintenance of the dendritic spines [8, 18] are thought to be dependent on Rab4 activity. The activity of Rab4 has also been proposed to regulate Rab4 associations with SNARE proteins such as Syntaxin 4 [39]. Similar to our observations with Rab4, loss of HTT perturbed the axonal motility of Rab11 [57] by decreasing Rab11 association with membranes [42]. While Rab11 activity was essential for its axonal transport [57], decreased Rab11 activity with impaired recycling of transferrin receptors was seen in HD fibroblasts, which were rescued by constitutively active Rab11 [43]. Although further investigation would be needed to isolate whether cellular activities at the synapses are directly dependent on Rab4 activity, in both of these scenarios, excess Rab4 perhaps may rescue HTT-mediated phenotypes via increased association of Rab4 with functional endogenous *Drosophila* htt-containing vesicle complexes promoting its axonal motility and increasing Rab4 activity at synapses. Conversely, excess Rab4 could also promote the trafficking of HTT-independent Rab4 vesicle complexes via rip11/Rab11-FIP5/kinesin-2 to increase Rab4 activity dependent functions at synapses. Indeed, Rab4 activity was previously shown to induce the transport of associated vesicles in cholinergic neurons [14]. Therefore, several overlapping mechanisms likely exist which restore synaptic and behavioral dysfunctions caused by pathogenic HTT.

Our observations indicate the importance of the axonal motility of a moving HTT-Rab4 vesicle complex in maintaining normal synaptic functions. Our previous findings validate the existence of Rab11 with HTT-Rab4 as we found that the axonal motility of Rab11 was also affected by HTT [57], that Rab11 and HTT co-migrate within axons in vivo [77], and that Rab11 is present with Rab4 vesicles in mouse and mammalian neurons (Figs. 2, 4, 5). Therefore, taken together our findings highlight Rab4 and the axonal endosomal pathway as a potential target that can be explored for early therapeutics prior to the onset of neuronal degeneration and behavioral defects observed in HD.

## Supplementary information

**Additional file 1.** Supplementary Information.

**Additional file 2.** Movie S1.

**Additional file 3.** Movie S2.

**Additional file 4.** Movie S3.

**Additional file 5.** Movie S4.

**Additional file 6.** Movie S5.

**Additional file 7.** Movie S6.

**Additional file 8.** Movie S7.

**Additional file 9.** Movie S8.

**Additional file 10.** Movie S9.

**Additional file 11.** Movie S10.

**Additional file 12.** Movie S11.

**Additional file 13.** Movie S12.

## Abbreviations

HTT: Huntingtin
HD: Huntington’s disease
BDNF: Brain-derived neurotrophic factor
HAP1: Huntingtin-associated protein 1
HIP1: Huntingtin-interacting protein 1
DIC: Dynein intermediate chain
KLC: Kinesin light chain
Rip11: Rab11-interacting protein
SYT1: Synaptotagmin 1
SYB: Synaptobrevin
SYX17: Syntaxin17
NMJ: Neuromuscular junction
VF: Vesicle fraction
IP: Immunoprecipitation
WT: Wildtype
iPSC: Induced pluripotent stem cells
iNeurons: Induced neurons
