# Supplementary material for: Excess Rab4 rescues synaptic and behavioral dysfunction caused by defective HTT-Rab4 axonal transport in Huntington’s disease
Source: Acta Neuropathol Commun. 2020 Jul 1;8:97. doi: 10.1186/s40478-020-00964-z (PMC7331280; doi:10.1186/s40478-020-00964-z)
Supplement: Supplementary file 1 — Additional file 1. Supplementary Information. [file 40478_2020_964_MOESM1_ESM.pdf]

Supplementary Information for

Excess Rab4 rescues synaptic and behavioral dysfunction caused by defective HTT-Rab4 axonal transport in Huntington's disease

Joseph A White II†[1], Thomas J Krzystek †[1], Hayley Hoffmar-Glennon[1], Claire Thant[1], Katherine Zimmerman[1], Gary Iacobucci[1], Julia Vail[2], Layne Thurston[1], Saad Rahman[1], Shermali Gunawardena*[1]

*correspondence: sg99@buffalo.edu

† Joseph A White II and Thomas J Krzystek contributed equally to this work.

[1] Department of Biological Sciences, The State University of New York at Buffalo, New York 14260, [2] Department of Biological Engineering, Cornell University, Ithaca, NY

Author e-mails: jdangerwhite@hotmail.com, thomaskr@buffalo.edu, hayleyhg@buffalo.edu, claireth@buffalo.edu, kzimmerm@buffalo.edu, garyiaco@buffalo.edu, jev52@cornell.edu, laynethu@buffalo.edu, snrahman@buffalo.edu, sg99@buffalo.edu.

Keywords: Huntingtin, Rab4, axonal transport, synaptic defects, behavioral deficits, *in vivo* imaging, *Drosophila*, iPSCs, Huntington's disease.

Running title: Huntingtin-Rab4 vesicle motility is disrupted in HD.

This PDF file includes:

Supplementary Figures S1- S9 and figure legends
Table S1: Key resources and reagents
Movie legends

Supplementary Figure and Movie legends:

Figure S1. Reduction of htt disrupts Rab4 motility and causes Rab4 accumulations in axons. (**a**) Arrows depict location of the cell bodies and synapses. Representative images from movies and corresponding kymographs from larvae expressing YFP-Rab4 or YFP-Rab4 with 70% reduction of htt (htt-RNAi). X axis=distance (µm), Y axis=time (s). Bar=10µm. (**b**) Quantification of duration weighted segmental velocity (µm/s) indicate significant decreases in both anterograde ($p<0.01$) and retrograde ($p<0.01$) YFP-Rab4 vesicle velocities with 70% reduction of htt. n=10. (**c**) Quantification analysis shows that anterograde and retrograde Rab4-vesicle run lengths (µm) are significantly decreased ($p<0.01, p<0.01$), but that neither pause frequencies (sec^{-1}) nor pause durations (sec) of vesicles are changed (ns) with 70% reduction of htt. n=10. (**d**) Representative images of larval segmental nerves expressing Rab4-mRFP or Rab4-mRFP with htt$^{+/-}$. Quantification of Rab4 bocks in larval nerves (#) reveal a significant increase ($p<0.001$) in axonal accumulations of Rab4-mRFP with a 50% genetic reduction of htt. n=5. Bar = 10um. (**e**) Representative images of larval segmental nerves expressing Rab4-mRFP that have been immunostained for Rab4 (Abcam, 1:100). Note that Rab4-mRFP (Red arrowhead) and endogenous Rab4 immunostaining (green arrowheads) show a high degree of co-localization (yellow arrowheads) in the merge panel. Quantification of the avg. co-localization by volume of Rab4-mRFP puncta with Rab4 antibody immunostaining reveals an 89.4% co-localization percentage. Pearson's correlation (ImageJ. Coloc2) of the representative image (R) was found to be 0.58, indicative of high confidence in co-localization. n=5. *ns=p>0.01, *p<0.01, **p<0.001, ***p<0.0001. S*tatistical significance was determined using the two-sample two-sided Student's t-test.

Figure S2. Reduction of htt causes Rab4 accumulations at synaptic boutons. (**a**) Representative images of NMJs from muscle 6/7 segment A4-5 of 3rd instar larvae from htt-RNAi (70% reduction) and htt$^{+/-}$ (50% reduction) stained with HRP-FITC. Bar=5µm. Quantification analysis shows no significant changes (ns) to the number of synaptic boutons (#) or to the avg. average bouton area (µm^{2}). However, the avg. synaptic length (µm) is significantly decreased compared to WT ($p<0.01$ htt-RNAi, $p<0.01$ htt$^{+/-}$). n=8. (**b**) Representative images from larvae expressing Rab4-mRFP alone or co-expressing Rab4-mRPF with either htt-RNAi or htt$^{+/-}$ that have been stained with HRP-FITC. Note that larvae expressing Rab4-mRFP alone show homogenous Rab4-mRFP within all boutons. However, boutons from larvae expressing Rab4-mRPF with either htt-RNAi or htt$^{+/-}$ show increased Rab4 staining. Bar=5µm. Quantification analysis of HRP intensity (AU) in larval NMJs show no changes (ns) in HRP intensity across all genotypes; however, quantification of Rab4-mRFP intensity (AU) revealed that larvae co-expressing Rab4-mRFP with either htt-RNAi ($p<0.01$) or htt$^{+/-}$ ($p<0.01$) showed significantly increased Rab4-mRFP intensity compared to larvae expressing Rab4-mRFP alone. When Rab4-mRFP intensity at NMJs was normalized to HRP intensity (AU), quantification revealed a similar significant increase in larvae co-expressing Rab4-mRFP with either htt-RNAi ($p<0.01$) or htt$^{+/-}$ ($p<0.001$) compared to larvae expressing Rab4-mRFP alone. AU=arbitrary units. n=8. *ns=p>0.01, *p<0.01, **p<0.001, ***p<0.0001.* Statistical significance was determined using the two-sample two-sided Student's t-test.

Figure S3. Reductions of klc, klp64d, or unc-104 cause Rab4 axonal blockages within larval axons. (**a**) Representative images of Rab4-mRFP larval nerves alone or in the context of 50% reduction of either klc, klp64d, or unc-104. Note the presence of Rab4 axonal blockages in larval axons (white arrowheads) in larvae co-expressing Rab4-mRFP with either klc$^{+/-}$, klp64d$^{+/-}$, or unc-104$^{+/-}$ bar=10µm. Quantification of the average number (#) of Rab4 blocks per larvae reveal that larvae expressing Rab4-mRFP in the context of 50% reduction of klc ($p<0.01$), klp64d ($p<0.001$), or unc-104 ($p<0.01$) show significant amounts Rab4-mRFP blocks compared to Rab4-mRFP expressing larvae (WT). n=5. (**b**) Sub-pixel immunofluorescence analysis revealed Rab4-mRFP (red arrows) and rip11-GFP (green arrows) co-localize (yellow arrows) within larval axons. Intensity-pixel plots show overlapping (purple arrows) peak intensities for Rab4 (red) and rip11 (green). n=5. *ns=p>0.01, *p<0.01, **p<0.001, ***p<0.0001.* Statistical significance was determined using the two-sample two-sided Student's t-test.

Figure S4. HTT15Q-mRFP motility within larval axons depends on endogenous htt. (**a**) Representative movies with kymographs from HTT15Q-mRFP larvae alone or in the context of 70% reduction of htt (htt-RNAi) or a 50% genetic reduction of htt (htt$^{+/-}$, [Df(98E2) CG9990]). Bar=10µm. Quantification indicates significant decreases in the duration weighted segmental anterograde HTT15Q-mRFP velocities with htt-RNAi ($p<0.00001$) or htt$^{+/-}$ ($p<0.0001$) and significant decreases in the duration weighted segmental retrograde HTT15Q-mRFP velocities with htt-RNAi ($p<0.001$) or htt$^{+/-}$ ($p<0.001$). (**b**) Quantification of Rab4-mRFP motility dynamics showed that 50% reduction of hip1 significantly decreased Rab4-mRFP retrograde run lengths ($p<0.01$) and retrograde pause frequencies ($p<0.01$), but had no effect (*ns*) on anterograde run lengths, anterograde pause frequencies, anterograde pause durations, or retrograde pause durations. n=10. (**c**) Quantification of HTT15Q-mRFP motility dynamics showed that 50% reduction

of hip1 significantly decreased HTT15Q-mRFP retrograde run lengths (*p<0.01*), but had no effect (*ns*) on anterograde run lengths, anterograde pause frequencies, retrograde pause frequencies, anterograde pause durations, or retrograde pause durations. n=10. *ns=p>0.01, *p<0.01, **p<0.001, ***p<0.0001, ****p<0.00001.* Statistical significance was determined using the two-sample two-sided Student's t-test.

Figure S5. mCherry-Rab4 motility is disrupted in iNeurons differentiated from HD patient iPSCs. (**a**) Representative images of HD (72Q) patient iPSCs stained with the pluripotent marker OCT4 (i). HD-patient NPCs stained with NESTIN (ii). HD-patient iPSCs differentiated to iNeurons stained with MAP2 and BIII-tubulin. Hoechst was used to stain nuclei. (**b-d**) Electrophysiological analysis of WT and HD patient-derived iNeurons. Note that these iNeurons elicit action potentials that are abolished in the presence of TTX. Electrophysiological analysis of WT and HD patient-derived iNeurons exhibit changes in Na+ and K+ currents that are sensitive to either TTX or TEA respectively. **(e)** Significant decreases in both the anterograde (*p<0.01*) and retrograde (*p<0.01*) run lengths of mCherry-Rab4 containing vesicles are seen. n=7. *ns=p>0.01, *p<0.01.* Statistical significance was determined using the non-parametric Wilcoxon–Mann–Whitney rank sum test.

Figure S6. Rab4 localizes with polyQ and HTT accumulations in HD iNeurons differentiated from HD patient iPSCs. (a) Representative image of an axonal blockage (arrow) containing HTT and Rab4 is seen in a neurite differentiated from HD-patient iPSC with 109Q repeats, using an antibody against HTT and Rab4. Intensity plots show overlapping peak (purple arrow). X=distance (µm), Y=intensity (AU). Bar=5µm. **(b)** Representative images from normal (WT) neurons derived from a normal individual (25Q) and HD iNeurons derived from a HD patient (72Q) stained with antibodies to Rab4 (green arrows), polyQ (red arrows) and Hoechst (blue). Co-localization of Rab4 and polyQ is seen in WT neurons (yellow arrows). Not all Rab4 puncta (green arrows) or polyQ puncta (red arrows) co-localize. Accumulations of Rab4 (green arrows) and polyQ (red arrows) are seen at the proximal end of the HD iNeurons. Only a few puncta show co-localization (yellow arrows). Bar=5µm. Intensity plots show overlapping peaks in WT iNeurons (purple arrows). Plots from HD iNeurons show red and green peaks with fewer overlapping peaks (purple). X=distance (µm), Y=intensity (AU). **(c,d)** Representative high-resolution images from WT iNeurons stained for Rab4 and DIC (**c**), Rab4 and HIP1 (**d**) show Rab4 co-localizes with DIC or HIP1 (yellow arrows). The HD iNeurons show Rab4 only (red arrows) or DIC or HIP1 only (green arrows) puncta (Bar = 5µm). Intensity plots show Rab4 and DIC or Rab4 and HIP1 overlapping puncta (purple arrows) in WT iNeurons, while in HD iNeurons only Rab4 (red) and DIC or HIP1 (green) puncta are seen. X=distance (µm), Y=intensity (AU).

Figure S7. The putative HTT-Rab4 synaptic complex is disrupted in HD iNeurons differentiated from HD patient iPSCs. (**a**) Analysis of total proteins from WT and HD iNeuronal culture homogenates using antibodies to HTT, Rab4, HIP1, DIC, KIF5C, Rab11-FIP5 and actin (loading control). Both full length (FL-HTT) and HTT fragments are observed in WT and HD iNeuronal cultures. Molecular weight ladder is indicated in Kd. Quantifications of band intensity on western blots (AU) revealed that DIC (*p<0.005*) and HIP1 (*p<0.005*) levels are significantly decreased in HD compared to WT. The level of KIF5C is also decreased in HD compared to WT although this was not significant (*ns*). The level of Rab4 and HTT show an increased trend in HD compared to WT. A trend towards increased levels is also seen for Rab11-FIP5 in HD compared to WT. Y axis=normalized intensity to WT (AU). Protein levels were normalized to actin and then to WT. n=3. *ns=p>0.05, *p<0.05, **p<0.005. S*tatistical significance was determined using the two-sample two-sided Student's t-test.

Figure S8. Expression of pathogenic HTT in *Drosophila* larval axons cause Rab4 blockages within larval axons and elevated Rab4 levels at NMJs. (**a**) Larvae expressing HTT103Q-eGFP and Rab4-mRFP show axonal blockages that contain both HTT and Rab4 (arrows). Bar= 5µm. (**b**) Expression of Rab4-mRFP with HTT16Q or HTT128Q does not affect the average number of axonal blocks (ns). n=8. (**c**) Representative images of NMJs from muscle 6/7 segment A4-5 in larvae expressing Rab4-mRFP alone or co-expressing Rab4-mRPF with either HTT16Q or HTT128Q stained with HRP-FITC. Note that larvae expressing Rab4-mRFP alone show homogenous Rab4-mRFP within all boutons, which is seemingly lost with HTT128Q. Bar=5µm. Quantification analysis of HRP intensity (AU) in larval NMJs show no changes (ns) in HRP intensity across all genotypes; however, quantification of Rab4-mRFP intensity (AU) revealed that larvae co-expressing Rab4-mRFP with either HTT128Q showed significantly increased Rab4-mRFP intensity compared to larvae expressing Rab4-mRFP alone (*p<0.0001*) or to larvae co-expressing Rab4-mRFP with HTT16Q (*p<0.001*). When Rab4-mRFP intensity at NMJs was normalized to HRP intensity (AU), quantification revealed a similar significant increase in larvae co-expressing Rab4-mRFP with HTT128Q compared to larvae expressing Rab4-mRFP alone (*p<0.001*) or to larvae co-expressing Rab4-mRFP with HTT16Q (*p<0.001*). Note that larvae expressing Rab4-mRFP alone and larvae co-expressing Rab4-mRPF with HTT16Q show unchanged intensities

at NMJs (ns). AU=arbitrary units. n=8. *ns=p>0.01, *p<0.01, **p<0.001, ***p<0.0001.* Statistical significance was determined using the two-sample two-sided Student's t-test.

Figure S9. A model of the putative moving HTT-Rab4 vesicle and how it is disrupted in HD. (**a**) Our observations propose a working model where a specific HTT-Rab4 vesicle complex that contains synaptic SNARE proteins synaptotagmin and synaptobrevin, and Rab11 is attached to molecular motors kinesin and dynein via HIP1 and is transported down axons for synaptic function. (**b**) In HD, polyQ-HTT present with this HTT-Rab4 vesicle disrupts associations between accessory proteins by decreasing the binding affinity of HIP1. Together, these aberrant associations dislocate the link between the polyQ-HTT-Rab4 vesicle and motor proteins kinesin-1 and dynein, resulting in the perturbation of its motility within axons causing synaptic and locomotion defects.

Movie S1
A representative movie from a larva expressing HTT25Q-eGFP (green) and Rab4-mRFP (red). Note that HTT and Rab4 co-migrate (yellow puncta) within larval axons.

Movie S2
A representative movie from a larva expressing Rab4-mRFP (red) and APP-YFP (green). Note that APP-YFP (green) and Rab4-mRFP (red) do not co-migrate within larval axons.

Movie S3
A representative movie from a larva expressing HTT-mRFP (red) and APP-YFP (green). Note that HTT-mRFP (red) and APP-YFP (green) co-migrate (yellow) within larval axons.

Movie S4
A representative movie from a larva expressing Rab4-mRFP (red) and nSyb-GFP (green). Note that nSyb-GFP (green) and Rab4-mRFP (red) co-migrate (yellow) within larval axons.

Movie S5
A representative movie from a larva expressing Rab4-mRFP (red) and Syt-GFP (green). Note that Syt-GFP (green) and Rab4-mRFP (red) co-migrate (yellow) within larval axons.

Movie S6
A representative movie from a larva expressing HTT15Q-mRFP (red) and nSyb-GFP (green). Note that nSyb-GFP (green) and HTT15Q-mRFP (red) co-migrate (yellow) within larval axons.

Movie S7
A representative movie from a larva expressing HTT15Q-mRFP (red) and Syt-GFP (green). Note that Syt-GFP (green) and HTT15Q-mRFP (red) co-migrate (yellow) within larval axons.

Movie S8
A representative movie from a larva expressing HTT15Q-mRFP (red) and Rab11-GFP (green). Note that Rab11-GFP (green) and Rab4-mRFP (red) co-migrate (yellow) within larval axons.

Movie S9
A representative movie from a larva expressing Rab4-mRFP(red) and Rab11-GFP (green). Note that Rab11-GFP (green) and Rab4-mRFP (red) co-migrate (yellow) within larval axons.

Movie S10
A representative movie from a larva expressing Rab4-mRFP (red) and YFP-Rab3 (green). Note that YFP-Rab3 (green) and Rab4-mRFP (red) do not co-migrate within larval axons.

Movie S11
A representative movie from a normal (WT) human iNeuron transfected with mCherry-Rab4 shows that Rab4 moves bi-directionally within the human neurite.

Movie S12

A representative movie from a HD patient iNeuron (Q72) transfected with mCherry-Rab4 shows that the motility of Rab4 is decreased.

Figure.S1

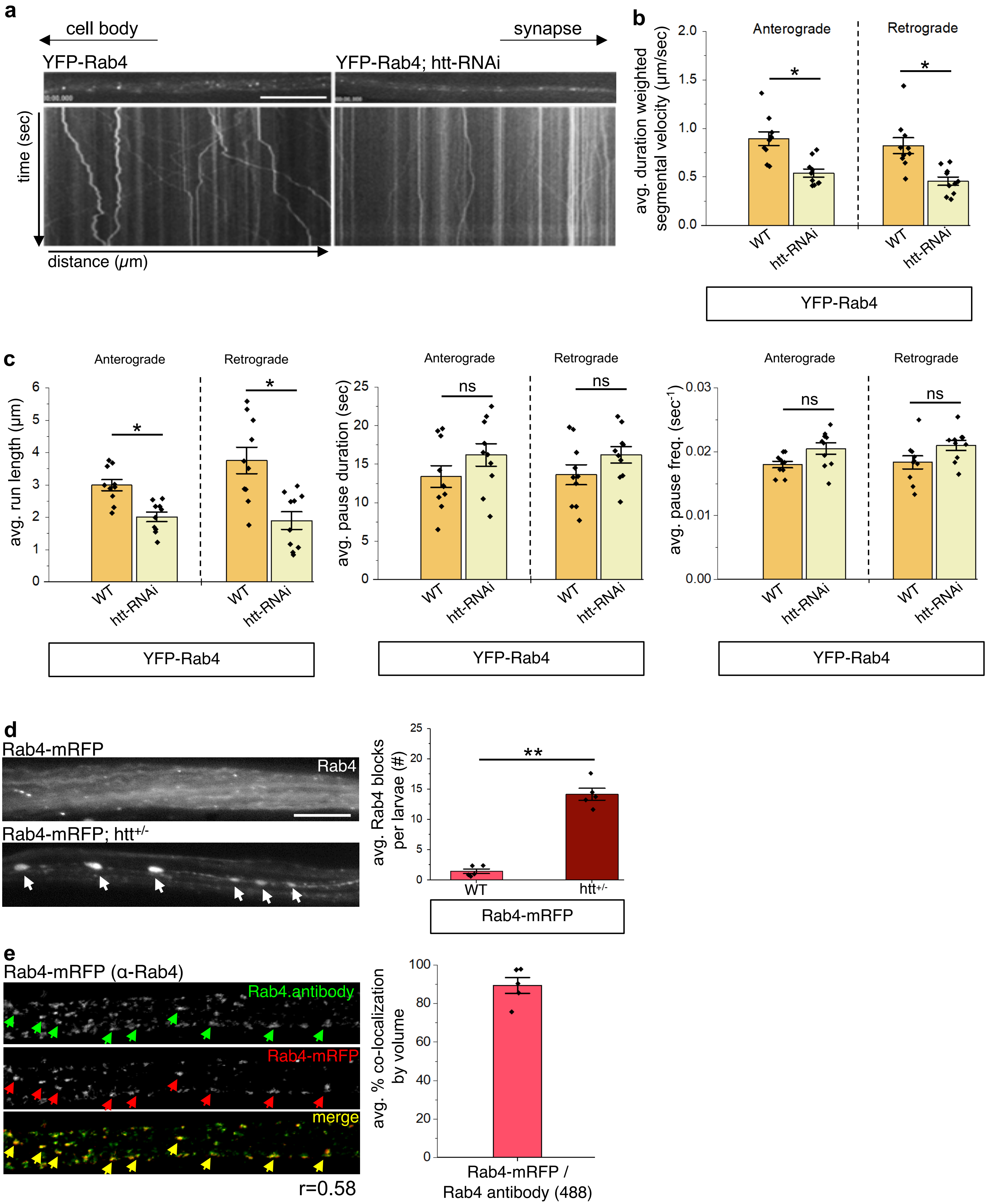

a
cell body
synapse
YFP-Rab4
YFP-Rab4; htt-RNAi
time (sec)
distance (μm)
b
Anterograde
Retrograde
avg. duration weighted segmental velocity (μm/sec)
WT
htt-RNAi
YFP-Rab4
c
avg. run length (μm)
avg. pause duration (sec)
avg. pause freq. (sec-1)
ns
d
Rab4-mRFP
Rab4
Rab4-mRFP; htt+/-
avg. Rab4 blocks per larvae (#)
htt+/-
e
Rab4-mRFP (α-Rab4)
Rab4.antibody
merge
r=0.58
avg. % co-localization by volume
Rab4-mRFP / Rab4 antibody (488)

Figure.S2

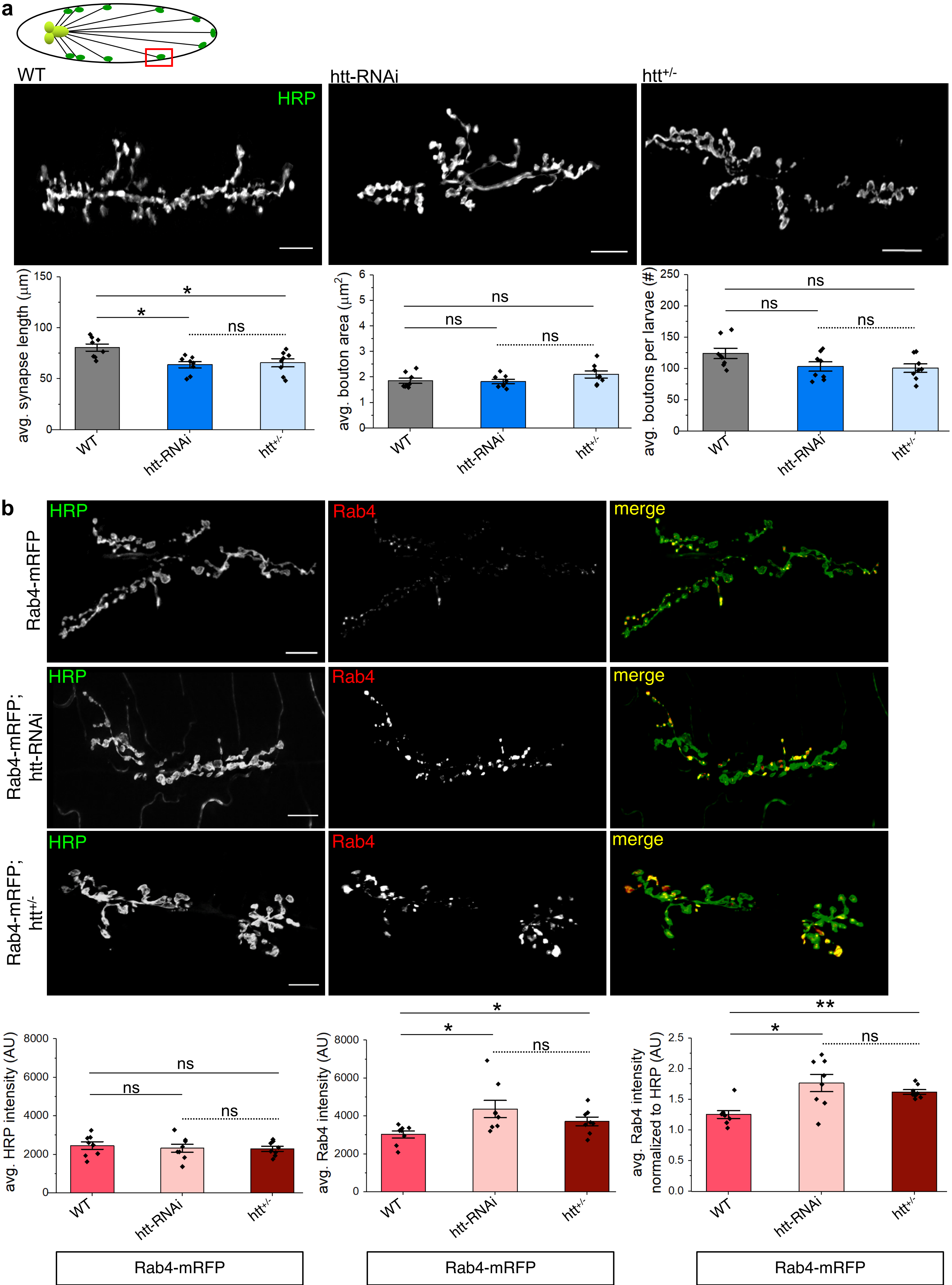

a
WT
htt-RNAi
htt+/-
HRP
avg. synapse length (μm)
avg. bouton area (μm²)
avg. boutons per larvae (#)
ns
*
WT
htt-RNAi
htt+/-
b
HRP
Rab4
merge
Rab4-mRFP
Rab4-mRFP; htt-RNAi
Rab4-mRFP; htt+/-
avg. HRP intensity (AU)
avg. Rab4 intensity (AU)
avg. Rab4 intensity normalized to HRP (AU)
**

Figure.S3

a

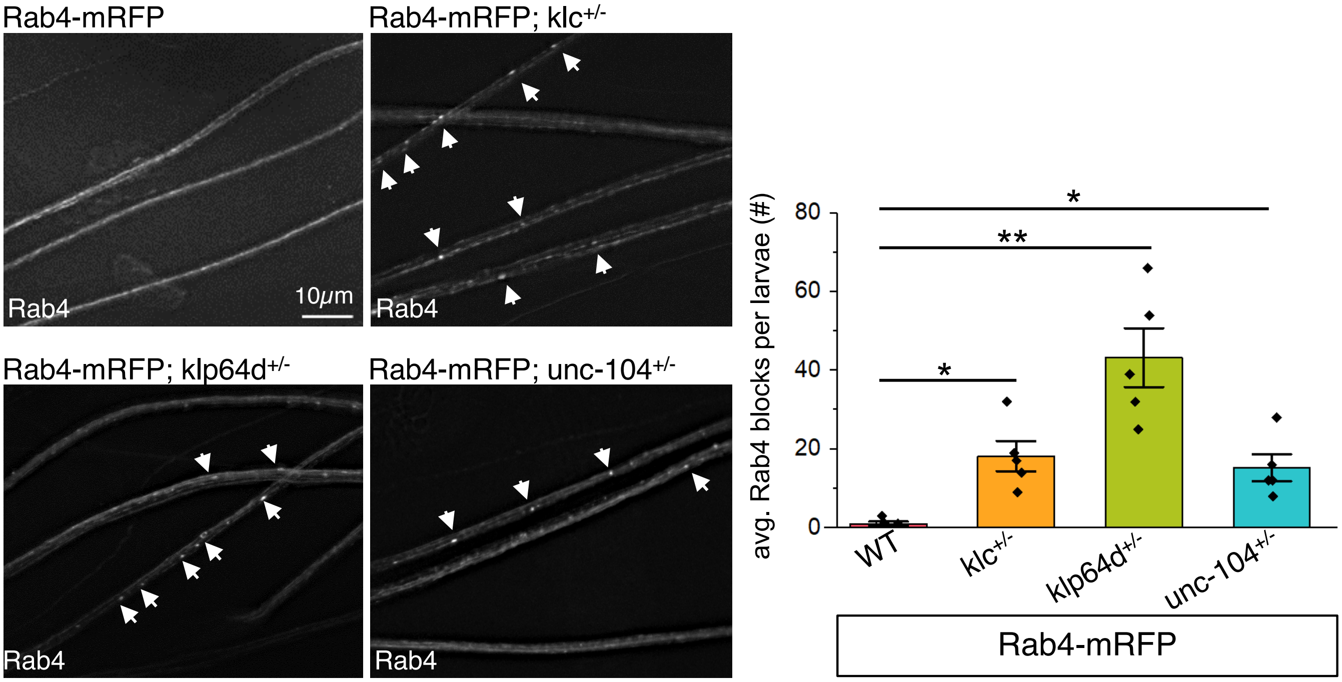
Rab4-mRFP
Rab4-mRFP; klc+/-
Rab4-mRFP; klp64d+/-
Rab4-mRFP; unc-104+/-
Rab4
10μm
avg. Rab4 blocks per larvae (#)
80
60
40
20
0
*
**
*
WT
klc+/-
klp64d+/-
unc-104+/-
Rab4-mRFP

b

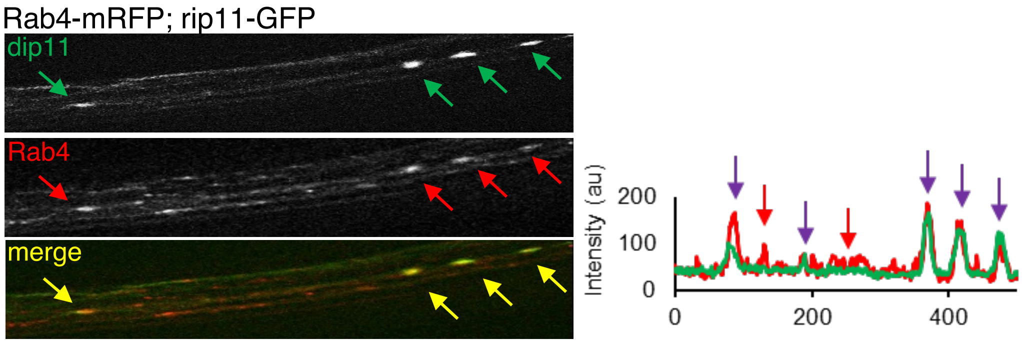
Rab4-mRFP; rip11-GFP
dip11
Rab4
merge
Intensity (au)
200
100
0
0
200
400

Figure.S4

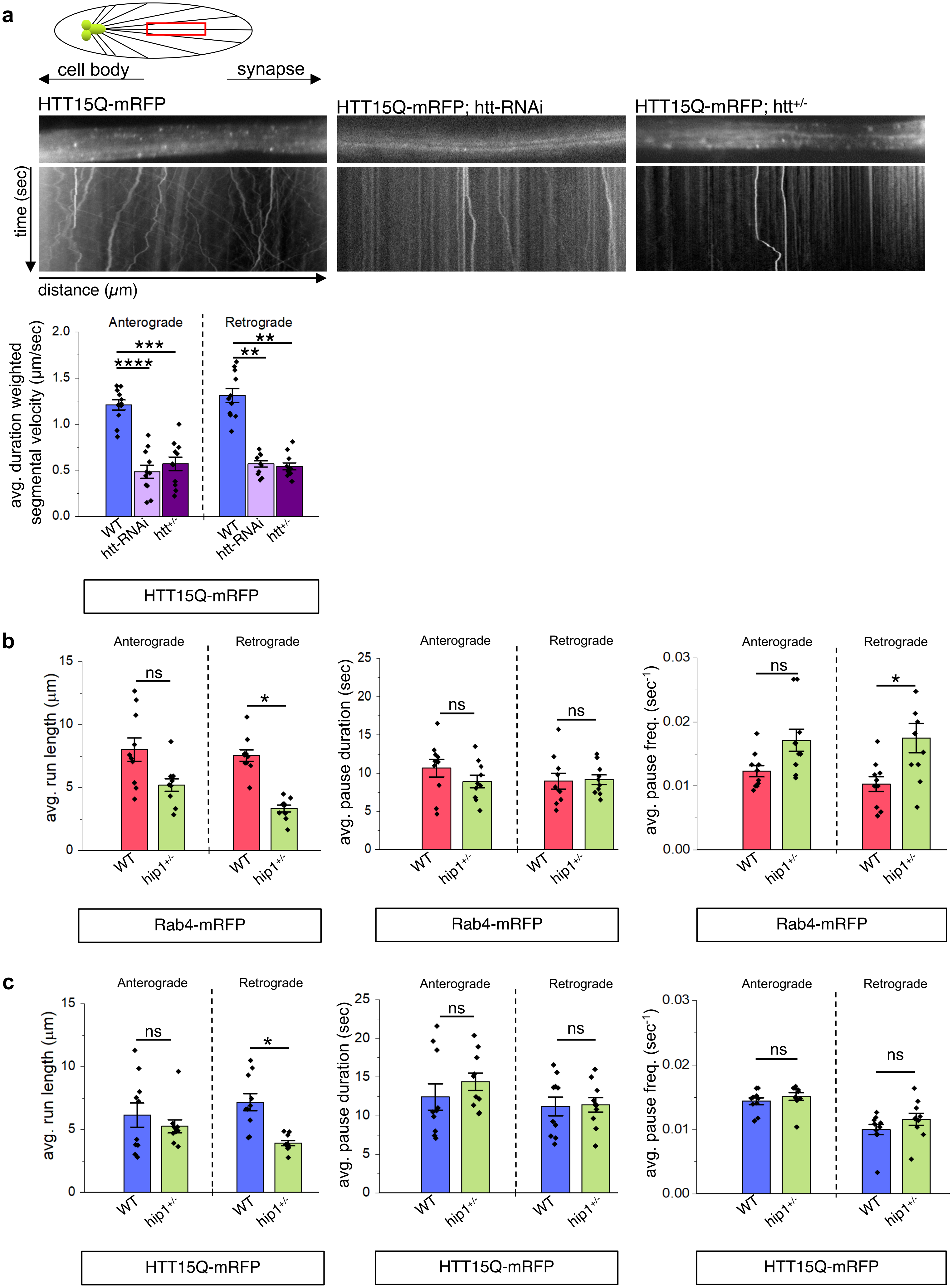
a
cell body
synapse
HTT15Q-mRFP
HTT15Q-mRFP; htt-RNAi
HTT15Q-mRFP; htt+/-
time (sec)
distance (μm)
Anterograde
Retrograde
avg. duration weighted segmental velocity (μm/sec)
WT
htt-RNAi
htt+/-
HTT15Q-mRFP
b
avg. run length (μm)
avg. pause duration (sec)
avg. pause freq. (sec-1)
ns
*
hip1+/-
Rab4-mRFP
c
HTT15Q-mRFP

Figure.S5

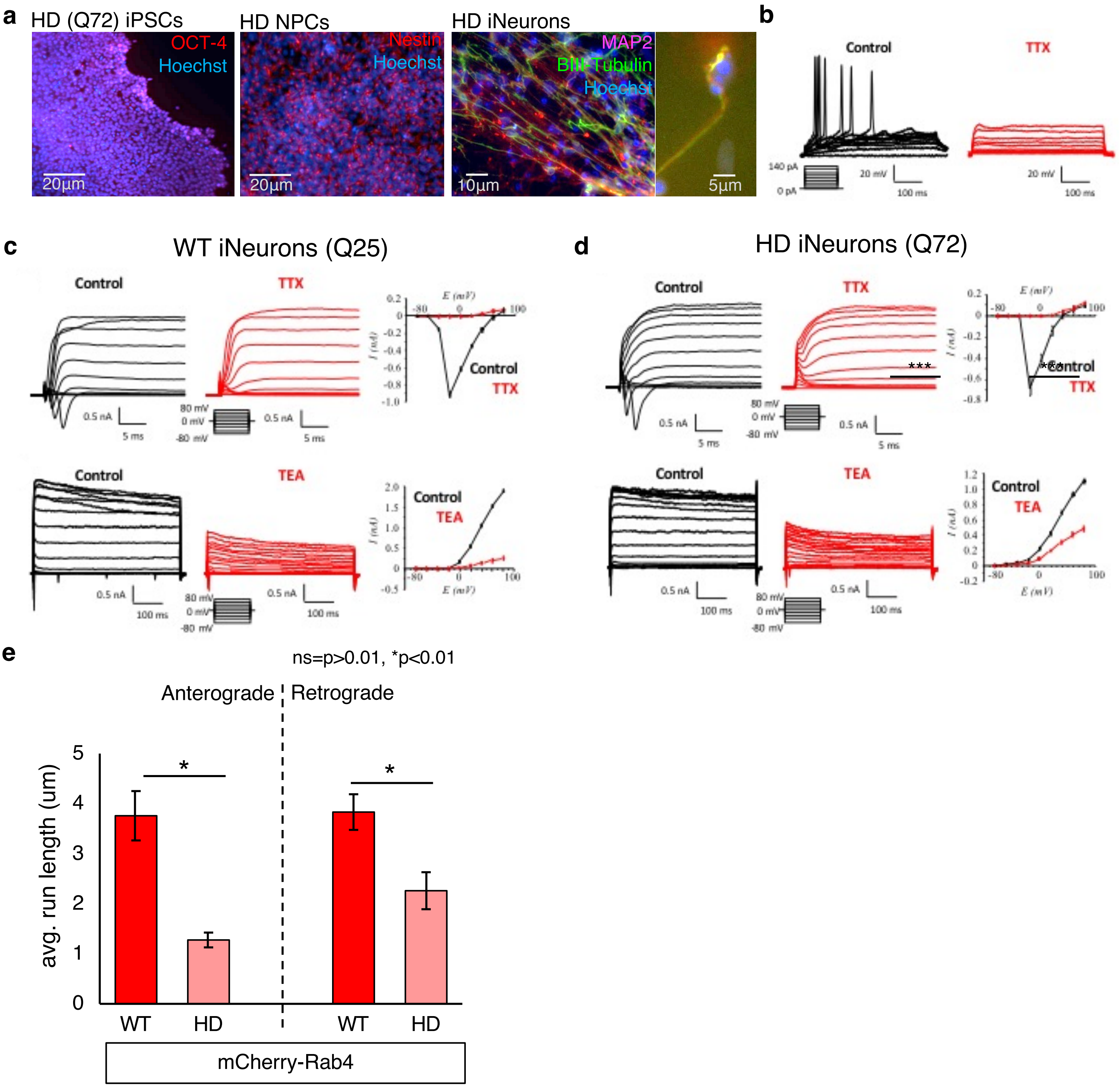

a
HD (Q72) iPSCs
OCT-4
Hoechst
20µm
HD NPCs
Nestin
Hoechst
20µm
HD iNeurons
MAP2
BIII-Tubulin
Hoechst
10µm
5µm
b
Control
TTX
140 pA
0 pA
20 mV
100 ms
c
WT iNeurons (Q25)
Control
TTX
0.5 nA
5 ms
80 mV
0 mV
-80 mV
E (mV)
I (nA)
Control
TTX
Control
TEA
0.5 nA
100 ms
d
HD iNeurons (Q72)
Control
TTX
***
Control
TTX
Control
TEA
0.5 nA
100 ms
e
ns=p>0.01, *p<0.01
Anterograde
Retrograde
avg. run length (um)
5
4
3
2
1
0
*
*
WT
HD
WT
HD
mCherry-Rab4

Figure.S6

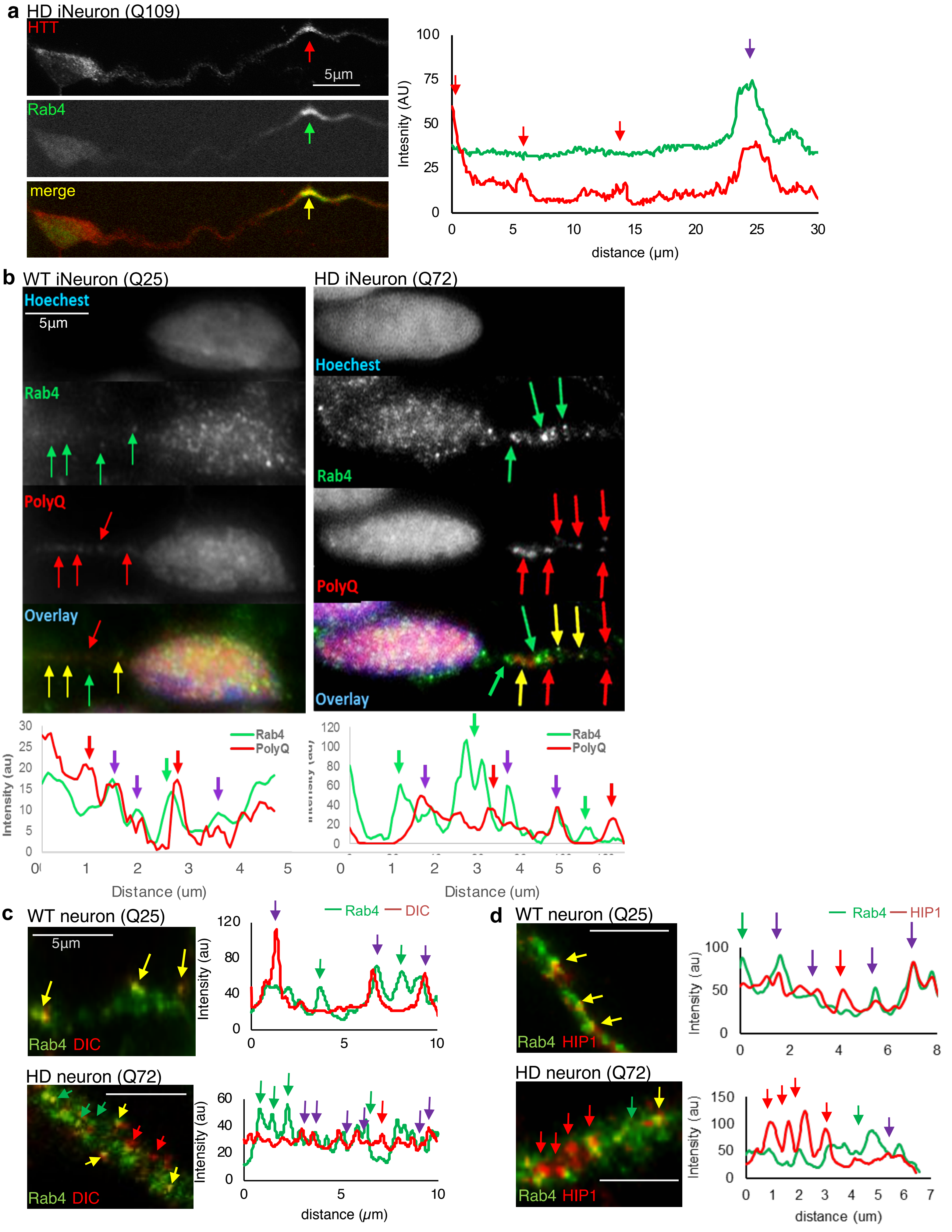
a
HD iNeuron (Q109)
HTT
5µm
Rab4
merge
Intesnity (AU)
distance (µm)
b
WT iNeuron (Q25)
HD iNeuron (Q72)
Hoechest
Rab4
PolyQ
Overlay
Rab4
PolyQ
Intensity (au)
Distance (um)
c
WT neuron (Q25)
Rab4 DIC
HD neuron (Q72)
Intensity (au)
distance (µm)
d
WT neuron (Q25)
Rab4 HIP1
HD neuron (Q72)
Intensity (au)
distance (um)

Figure.S7

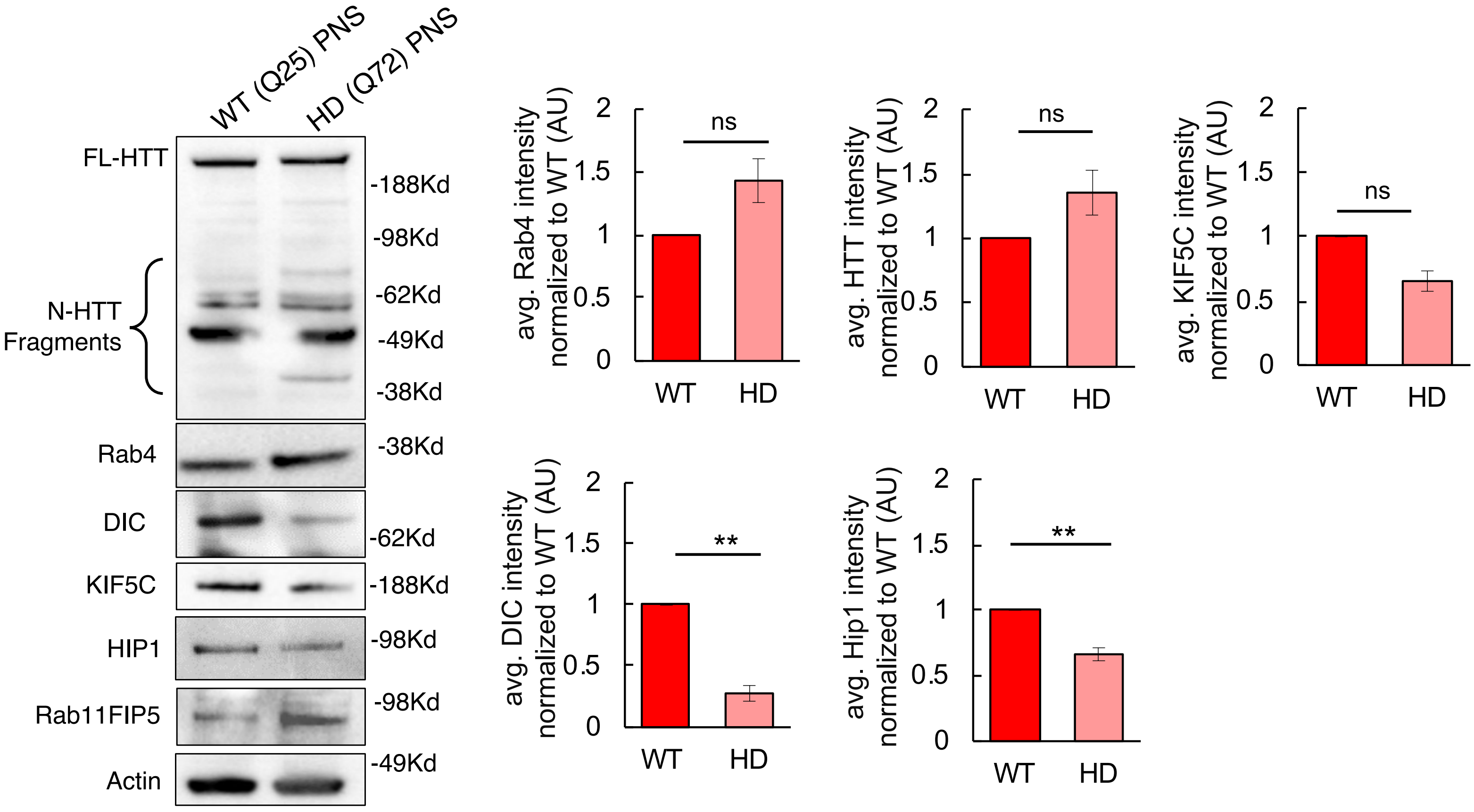
WT (Q25) PNS
HD (Q72) PNS
FL-HTT
N-HTT Fragments
Rab4
DIC
KIF5C
HIP1
Rab11FIP5
Actin
-188Kd
-98Kd
-62Kd
-49Kd
-38Kd
avg. Rab4 intensity normalized to WT (AU)
avg. HTT intensity normalized to WT (AU)
avg. KIF5C intensity normalized to WT (AU)
avg. DIC intensity normalized to WT (AU)
avg. Hip1 intensity normalized to WT (AU)
ns
**
WT
HD

Figure.S8

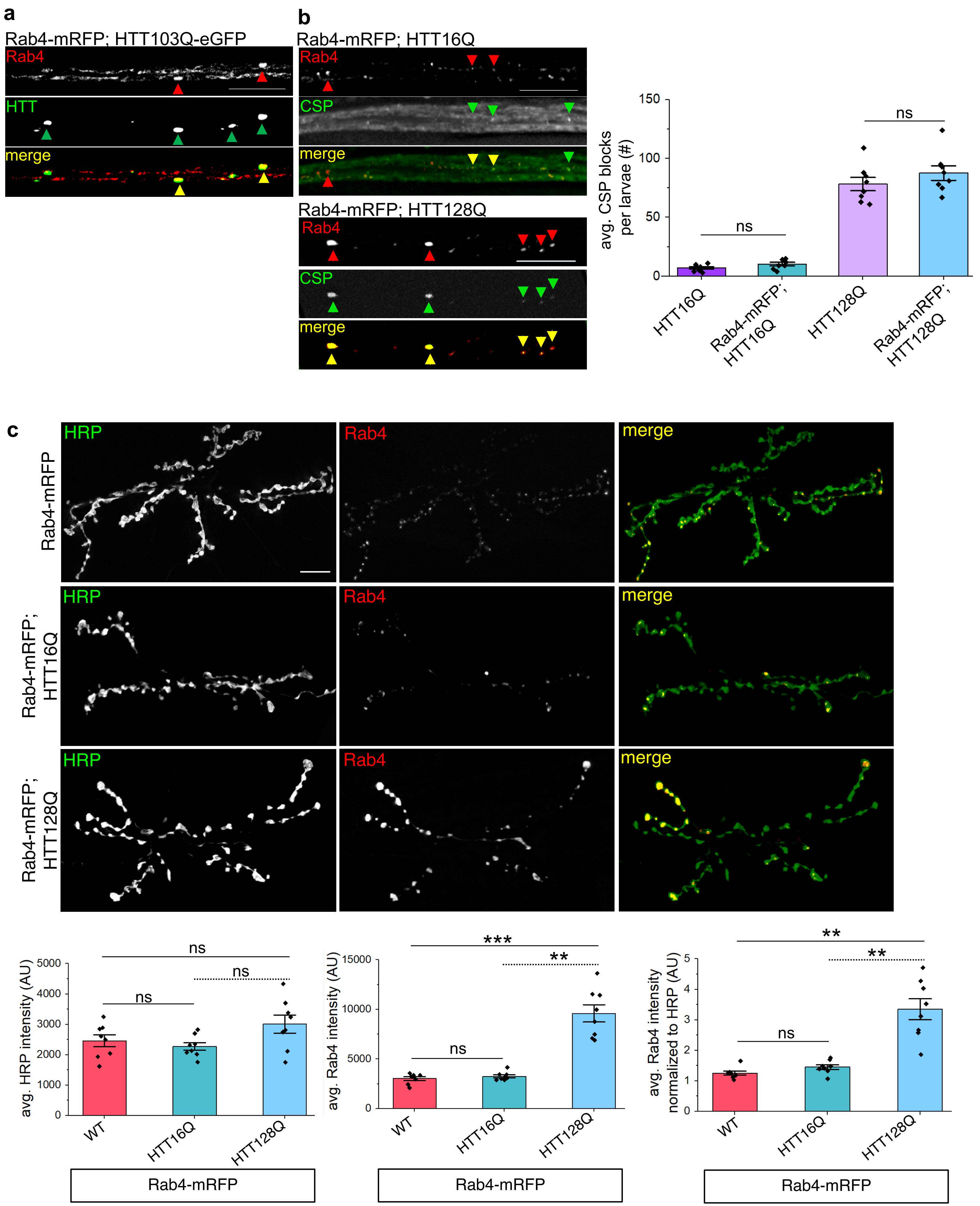
a
Rab4-mRFP; HTT103Q-eGFP
Rab4
HTT
merge
b
Rab4-mRFP; HTT16Q
Rab4
CSP
merge
Rab4-mRFP; HTT128Q
Rab4
CSP
merge
avg. CSP blocks per larvae (#)
150
100
50
0
ns
ns
HTT16Q
Rab4-mRFP; HTT16Q
HTT128Q
Rab4-mRFP; HTT128Q
c
Rab4-mRFP
Rab4-mRFP; HTT16Q
Rab4-mRFP; HTT128Q
HRP
Rab4
merge
avg. HRP intensity (AU)
5000
4000
3000
2000
1000
0
ns
ns
ns
WT
HTT16Q
HTT128Q
Rab4-mRFP
avg. Rab4 intensity (AU)
15000
10000
5000
0
***
**
ns
avg. Rab4 intensity normalized to HRP (AU)
5
4
3
2
1
0
**
**
ns

Figure.S9

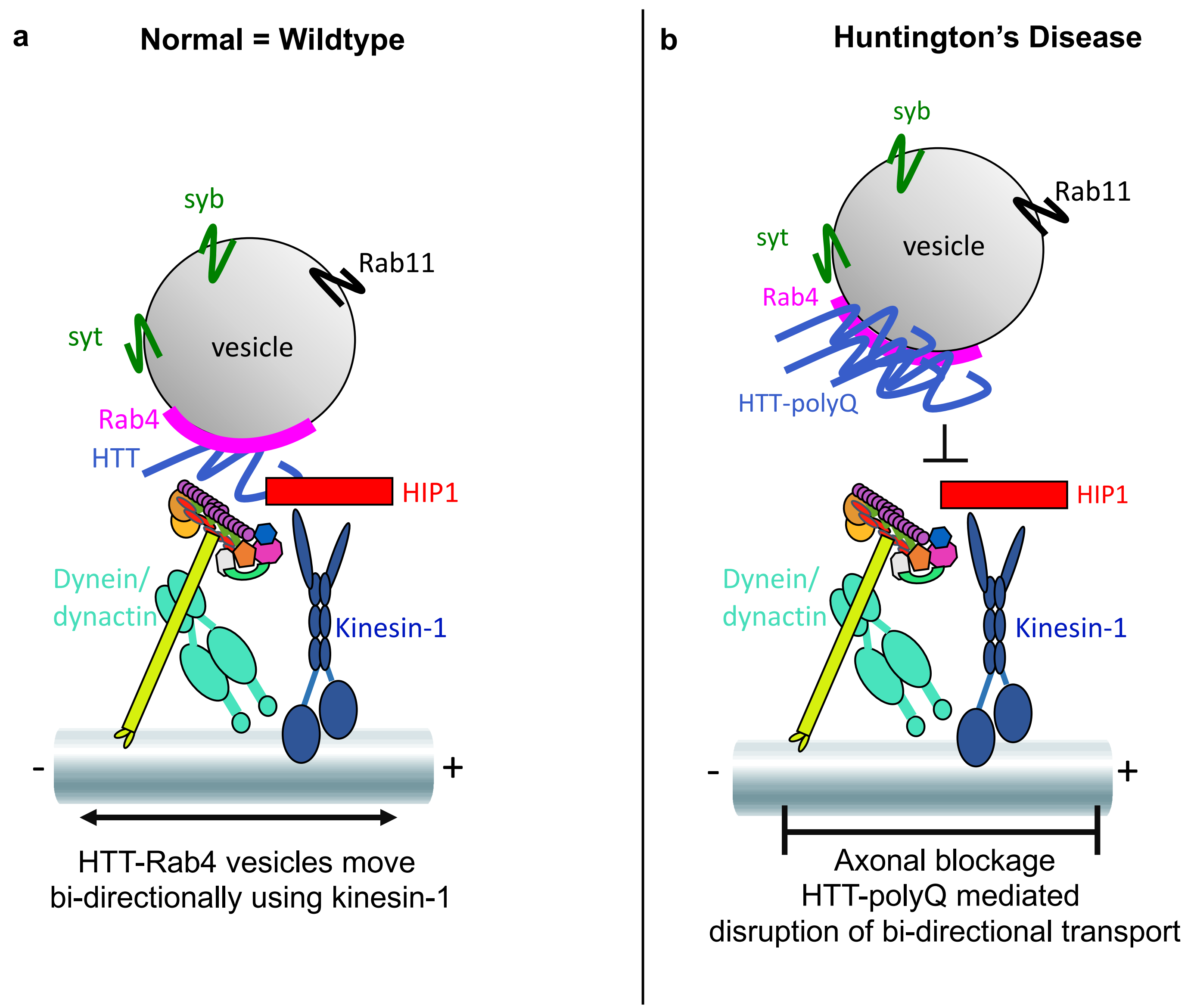
a
Normal = Wildtype
syb
Rab11
syt
vesicle
Rab4
HTT
HIP1
Dynein/
dynactin
Kinesin-1
-
+
HTT-Rab4 vesicles move
bi-directionally using kinesin-1
b
Huntington's Disease
syb
Rab11
syt
vesicle
Rab4
HTT-polyQ
HIP1
Dynein/
dynactin
Kinesin-1
-
+
Axonal blockage
HTT-polyQ mediated
disruption of bi-directional transport

Table S1: Key resources and reagents

RESOURCE	SOURCE	IDENTIFIER
**Antibodies and Dyes**		
Rabbit anti-klc	Laboratory of Lawrence Goldstein	Gindhart et. al., 1996
Mouse anti-DIC (74.1)	Abcam	Cat# ab23905 RRID: AB_2096669
Mouse anti-OCT-3/4 (C-10)	Santa Cruz	Cat# sc-5279 RRID: AB_628051
Mouse anti-Nestin	Santa Cruz	Cat# sc-23927 RRID: AB_627994
Mouse anti-MAP2	BD Biosciences	Cat# 556320 RRID: AB_396359
Mouse anti-βIII-tubulin (TUBB3)	Biolegend	Cat# 801212 RRID: AB_2721321
Rabbit anti-Tyrosine Hydroxylase	EMD Millipore	Cat# ab152 RRID: AB_390204
Rabbit anti-Rab4 (monoclonal)	Abcam	Cat# ab109009 RRID: AB_10887396
Rabbit anti-Rab4 (polyclonal)	Abcam	Cat# ab13252 RRID: AB_2269374
Mouse anti-HIP1 (4B10)	Novus biological	Cat# NB300-203 RRID: AB_10000880
Mouse anti-PolyQ (MW1)	EMD Millipore	Cat# MABN2427 RRID: N/A
Rabbit anti-SYT1	Phosphosolutions	Cat# 1975-STG RRID: AB_2492251
Mouse anti-SYP (SY38)	Thermofisher	Cat# MAB5258 RRID: AB_2313839
Mouse anti-KIF5C	Laboratory of Lawrence Goldstein	Xia et. al., 1998
Rabbit anti-Actin	Thermofisher	Cat# MA5-32479 RRID: AB_2809756
Mouse anti-Tubulin (DM1A)	Abcam	Cat# ab7291 RRID: AB_2241126
Mouse anti-HTT (1HU-4C8)	EMD Millipore	Cat# MAB2166 RRID: AB_2123255
Rabbit anti-HTT (EP867Y)	Abcam	Cat# ab45169 RRID: AB_733062
Mouse anti-KDEL (10C3)	Abcam	Cat# ab12223 RRID: AB_298945
Mouse anti-Golgi (7H6D7C2)	Millipore Sigma	Cat# 345867 RRID: AB_564660
Mouse anti-Cytochrome C (A-8)	Santa Cruz	Cat# sc-13156 RRID: AB_627385
Rabbit anti-Rab11-FIP5	Thermofisher	Cat# PA5-31790 RRID: AB_2549263
Mouse anti-HAP1 (C-3)	Santa Cruz	Cat# sc-166245 RRID: AB_2116129
Rabbit anti-Rab11	Abcam	Cat# ab3612 RRID: AB_10861613
Rabbit anti-Rab5	Abcam	Cat# ab18211 RRID: AB_470264
Rat anti-Syntaxin17	Laboratory of Gabor Juhasz	Takats et. al., 2013

Table S1: Key resources and reagents

Mouse anti-DCSP-3 (1G12)	Developmental Studies Hybridoma Bank	Cat# DCSP-3 (1G12)<br>RRID: AB_528184
Anti-Mouse Alexa Fluor® 488	Thermofisher	Cat# A11001<br>RRID: AB_2534069
Anti-Mouse Alexa Fluor® 568	Thermofisher	Cat# A11004<br>RRID: AB_2534072
Anti-Mouse Alexa Fluor® 647	Thermofisher	Cat# A21235<br>RRID: AB_2535804
Anti-Rabbit Alexa Fluor® 488	Thermofisher	Cat# A11008<br>RRID: AB_143165
Anti-Rabbit Alexa Fluor® 568	Thermofisher	Cat# A11011<br>RRID: AB_143157
Anti-Rabbit Alexa Fluor® 647	Thermofisher	Cat# A21244<br>RRID: AB_141663
Anti-Mouse secondary antibody, HRP	Thermofisher	Cat# 32430<br>RRID: AB_1185566
Anti-Rabbit secondary antibody, HRP	Thermofisher	Cat# 32460<br>RRID: AB_1185567
Alexa Fluor® 594 Goat Anti-Horseradish Peroxidase	Jackson ImmunoResearch Labs	Cat# 123-585-021<br>RRID: AB_2338966
Fluorescein (FITC) Goat Anti-Horseradish Peroxidase	Jackson ImmunoResearch Labs	Cat# 123-095-021<br>RRID: AB_2314647
Hoechst	Thermofisher	Cat# H3570<br>RRID: AB_10626776
**Bacterial and Virus Strains**		
mCherry-Rab4a-7	Laboratory of Michael Davidson<br><br>De Franceschi et. al., 2016	Addgene<br>Plasmid # 55125<br>RRID: Addgene_55125
**Biological Samples**		
Mouse brain tissue (C57BL/6)	Laboratory of Kathryn Medler	Jackson Laboratory, Stock No:000664
**Chemicals, Peptides, and Recombinant Proteins**		
Lipofectamine 3000	Fisher	Cat# L3000015<br>RRID: N/A
Corning Matrigel	Fisher	Cat# CB40230A<br>RRID: N/A
Advanced DMEM/F12	Invitrogen	Cat# 12634028<br>RRID: N/A
Essential 8 media	Invitrogen	Cat# A1517001<br>RRID: N/A
Neurobasal media	Invitrogen	Cat# 21103049<br>RRID: N/A
PSC neural induction media	Invitrogen	Cat# A1647801<br>RRID: N/A
B27 supplement media	Invitrogen	Cat# 17504-044<br>RRID: N/A

Table S1: Key resources and reagents

Protease inhibitor cocktail	Pierce	Cat# PIA32965<br>RRID: N/A
Phosphatase Inhibitor	Pierce	Cat# PI88667<br>RRID: N/A
Protein A/G Magnetic Beads	Pierce	Cat# PI88802<br>RRID: N/A
Vecta Shield Mounting Medium	Fisher	Cat# NC9265087<br>RRID: N/A
**Experimental Models: Human Cell Lines**		
GM23279, polyQ=25, 26y, female	NIGMS Repository (Coriell Institute for Medical Research - Camden, NJ)	Cat# GM23279<br>RRID: CVCL_F178
GM23225, polyQ=72, 20y, female	NIGMS Repository (Coriell Institute for Medical Research - Camden, NJ)	Cat# GM23225<br>RRID: CVCL_F169
ND38555, polyQ=17, 48y, female	NINDS Repository (Coriell Institute for Medical Research - Camden, NJ)	Cat# ND3855<br>RRID: CVCL_Y822
ND42222, polyQ=109, 9y, female	NINDS Repository (Coriell Institute for Medical Research - Camden, NJ)	Cat# ND42222<br>RRID: CVCL_Y844
**Experimental Models: *D. melanogaster* Organisms/Strains**		
P{Appl-GAL4.G1a}1, y_{1} w_{*}	Bloomington Drosophila Stock Center	BDSC: 32040;<br>FlyBase: FBst0032040
Appl-GAL4; T(2,3), CyO, TM6B, Tb_{1} / Pin_{88k}	Laboratory of Lawrence Goldstein	Gunawardena & Goldstein, 2001
w_{*}; P{UAS-Rab4-mRFP}2	Bloomington Drosophila Stock Center	BDSC: 8505;<br>FlyBase: FBtst0008505
y_{1} w_{*}; P{UASp-YFP.Rab4}09	Bloomington Drosophila Stock Center	BDSC: 23269;<br>FlyBase: FBst0023269
UAS-htt-RNAi (UAS-dHTT-RNAi)	Laboratory of Lawrence Goldstein	Gunawardena et. al., 2003
Df(98E2); CG9990 / TM3 ($htt_{+/-}$)	Laboratory of Norbert Perrimon	Zhang et. al., 2009
y_{1} w_{*}; Mi{MIC}$Rab4_{MI10530}$	Bloomington Drosophila Stock Center	BDSC: 55497;<br>FlyBase: FBst0055497
pUAST-HTT15Q-mRFP	Laboratory of Troy Littleton	Weiss et. al., 2012
pUAST-HTTex1-25Q-eGFP	Laboratory of Norbert Perrimon	Zhang et. al., 2010
y_{1} w_{*}; P{UAS-APP.YFP}LG	Bloomington Drosophila Stock Center	BDSC: 32039;<br>FlyBase: FBst0032039
w_{*}; P{UAS-Syt-eGFP}3	Bloomington Drosophila Stock Center	BDSC: 6926;<br>FlyBase: FBst0006926
w_{*}; P{GawB}D42, P{UAS-nSyb-GFP.E}3/TM3, Sb_{1}	Bloomington Drosophila Stock Center	BDSC: 9263;<br>FlyBase: FBst0009263
y_{1} w_{*}; P{UASp-YFP.Rab3} $lolal_{02}$	Bloomington Drosophila Stock Center	BDSC: 9762;<br>FlyBase: FBst0009762

Table S1: Key resources and reagents

w_{*}; P{UAS-Rab11-GFP}2	Bloomington Drosophila Stock Center	BDSC: 8506; FlyBase: FBst0008506
klc_{8ex94} / TM6B	Laboratory of Lawrence Goldstein	Gindhart et. al., 1998
$robl_{K}$ / B3	Laboratory of Lawrence Goldstein	Bowman et. al., 1999
y_{1} w_{1}; $klp64D_{k1}$ / TM3, y_{+} Ser_{1}	Bloomington Drosophila Stock Center	BDSC: 5578; FlyBase: FBst0005578
w_{1118}; P{XP}$unc\text{-}104_{d11204}$ / CyO	Bloomington Drosophila Stock Center	BDSC: 19346; FlyBase: FBst0019346
y_{1} w_{67c23}; P{lacW}$milt_{k04704}$ / CyO	Bloomington Drosophila Stock Center	BDSC: 10553; FlyBase: FBst0010553
w_{1118}; Mi{ET1}$hip1_{MB04365}$	Bloomington Drosophila Stock Center	BDSC: 24809; FlyBase: FBst0024809
y_{1} w_{*}; P{lacW}nmo_{P1} / TM3, Sb_{1}	Bloomington Drosophila Stock Center	BDSC: 27897; FlyBase: FBst0027897
w_{1118}; Mi{ET1}$nuf_{MB09772}$	Bloomington Drosophila Stock Center	BDSC: 27803; FlyBase: FBst0027803
y_{1} P{SUPor-P}$rip11_{KG02485}$ / FM7c, sn_{+}	Bloomington Drosophila Stock Center	BDSC: 13742; FlyBase: FBst0013742
pUAST-rip11-GFP.CT	Laboratory of Donald Ready	Li et. al., 2007
w_{1118}; P{UAS-HTT.16Q.FL}F24 / CyO	Bloomington Drosophila Stock Center	BDSC: 33810; FlyBase: FBst0033810
w_{1118}; P{UAS-HTT.128Q.FL}f27b	Bloomington Drosophila Stock Center	BDSC: 33808; FlyBase: FBst0033808
pUAST-HTTex1-72Q-eGFP	Laboratory of Norbert Perrimon	Zhang et. al., 2010
pUAST-HTTex1-103Q-eGFP	Laboratory of Norbert Perrimon	Zhang et. al., 2010
pUAST-HTT138Q-mRFP	Laboratory of Troy Littleton	Weiss et. al., 2012
**Software / Algorithms**		
MATLAB-based particle tracking program	Laboratories of Gaudenz Danuser and Lawrence Goldstein	Yang et. al., 2005, Reis et al 2012, Gunawardena et al 2013
ImageJ	Schneider et. al., 2012 https://www.imagej.net/	RRID: SCR_003070
Metamorph / Metavue Imaging Software	Molecular Devices, Sunnyvale, CA, USA	RRID: SCR_002368
Minitab18	https://www.minitab.com/en-us/	RRID: SCR_014483
Microsoft Excel	https://www.microsoft.com/en-gb/	RRID: SCR_016137
RStudio	http://www.rstudio.com/	RRID:SCR_000432
OriginLab / OriginPro	https://www.originlab.com/	RRID: SCR_014212
